# Zoonotic origin of the human malaria parasite *Plasmodium malariae* from African apes

**DOI:** 10.1038/s41467-022-29306-4

**Published:** 2022-04-06

**Authors:** Lindsey J. Plenderleith, Weimin Liu, Yingying Li, Dorothy E. Loy, Ewan Mollison, Jesse Connell, Ahidjo Ayouba, Amandine Esteban, Martine Peeters, Crickette M. Sanz, David B. Morgan, Nathan D. Wolfe, Markus Ulrich, Andreas Sachse, Sébastien Calvignac-Spencer, Fabian H. Leendertz, George M. Shaw, Beatrice H. Hahn, Paul M. Sharp

**Affiliations:** 1 Institute of Evolutionary Biology and Centre for Immunity, Infection and Evolution, University of Edinburgh, Edinburgh, EH9 3FL UK; 2 Department of Medicine, University of Pennsylvania, Philadelphia, PA 19104 USA; 3 Department of Microbiology, University of Pennsylvania, Philadelphia, PA 19104 USA; 4 Recherche Translationnelle Appliquée au VIH et aux Maladies Infectieuses, Institut de Recherche pour le Développement, University of Montpellier, INSERM, 34090 Montpellier, France; 5 Department of Anthropology, Washington University in St. Louis, St Louis, MO 63130 USA; 6 Wildlife Conservation Society, Congo Program, BP, 14537 Brazzaville, Republic of the Congo; 7 Lester E. Fisher Center for the Study and Conservation of Apes, Lincoln Park Zoo, Chicago, IL USA; 8 Metabiota Inc, San Francisco, CA 94117 USA; 9 Robert Koch Institute, 13353 Berlin, Germany; 10 Helmholtz Institute for One Health, Greifswald, Germany

**Keywords:** Parasite evolution, Parasite genomics, Genome informatics, Malaria

## Abstract

The human parasite *Plasmodium malariae* has relatives infecting African apes (*Plasmodium rodhaini*) and New World monkeys (*Plasmodium brasilianum*), but its origins remain unknown. Using a novel approach to characterise *P. malariae*-related sequences in wild and captive African apes, we found that this group comprises three distinct lineages, one of which represents a previously unknown, highly divergent species infecting chimpanzees, bonobos and gorillas across central Africa. A second ape-derived lineage is much more closely related to the third, human-infective lineage *P. malariae*, but exhibits little evidence of genetic exchange with it, and so likely represents a separate species. Moreover, the levels and nature of genetic polymorphisms in *P. malariae* indicate that it resulted from the zoonotic transmission of an African ape parasite, reminiscent of the origin of *P. falciparum*. In contrast, *P. brasilianum* falls within the radiation of human *P. malariae*, and thus reflects a recent anthroponosis.

## Abstract (web summary)

*Plasmodium malariae* is a cause of malaria in humans and related species have been identified in non-human primates. Here, the authors use genomic analyses to establish that human *P. malariae* arose from a host switch of an ape parasite whilst a species infecting New World monkeys can be traced to a reverse zoonosis.

## Introduction

*Plasmodium malariae* is one of six *Plasmodium* species that commonly cause malaria in humans^1^. Although geographically widespread across tropical and subtropical malaria-endemic regions^2,3^, *P. malariae* is the least well characterised human parasite, in part because it reaches only very low levels of parasitaemia, is generally associated with mild or no disease^4^, and frequently co-exists with other malaria parasites in multi-species infections^5^. Unlike other human parasites, *P. malariae* infects only mature red blood cells, with an intra-erythrocytic cycle of 72 h, which causes the characteristic quartan fever^4^. However, the parasite can also persist chronically and recrudesce years or decades after the initial infection^2,3^. Because of a lack of systematic studies due to the perceived low disease burden, the prevalence of *P. malariae* is likely underestimated^6–8^.

While the origins of the major human malaria parasites *P. falciparum* and *P. vivax* have recently been traced to parasites infecting African apes^9–15^, the evolutionary history of *P. malariae* remains unknown, despite the long-known existence of close relatives in non-human primates^16^. A quartan parasite morphologically indistinguishable from *P. malariae* was observed in the early 20^th^ century infecting wild chimpanzees, and given the name *P. rodhaini*^17^. However, in subsequent transmission studies *P. rodhaini* was successfully transferred from chimpanzees to humans by blood inoculation^18^, while *P. malariae* was transmitted from humans to chimpanzees by blood^19^ and infective mosquitoes^20^. This apparent lack of host specificity suggested that ape and human parasites represented members of the same species^16^, but this could not be verified because strains of *P. rodhaini* were not maintained and thus were never genetically characterised.

More recently, genetic studies have been applied to ape-infecting relatives of *P. malariae*. First, analyses of mitochondrial DNA (mtDNA) sequences derived from faecal samples from wild-living chimpanzees, gorillas and bonobos indicated the existence of two distinct *P. malariae*-related lineages infecting apes: one that is closely related to the human parasite and another that is substantially more divergent^9,21,22^. Echoing this, mtDNA sequences obtained from samples of mosquitoes (*Anopheles vinckei*) that feed on apes also seemed to fall into two divergent lineages^23^. Recently, genome sequences, one near-complete (PmlGA01) and one partial (PmlGA02), were recovered from the blood of two chimpanzees living at a wildlife reserve in Gabon^24^. Although genome-wide analyses revealed a close relationship to *P. malariae*, these “*P. malariae*-like” parasites were considered a separate species, that might have diverged from *P. malariae* more than 3 million years ago^24^. However, PmlGA01 and PmlGA02 were as divergent from one another as they were from *P. malariae*, and the study provided no information as to the relationship of these two parasites to the two divergent lineages of ape parasites revealed by mtDNA.

Finally, another *P. malariae*-related lineage, *P. brasilianum*, infects New World monkeys throughout Central and South America. Molecular characterisation of *P. brasilianum*, albeit at only a limited number of genetic loci, found it to be near-identical to *P. malariae*, again suggesting that the two lineages may in fact be a single species^4,24–26^. In addition, *P. brasilianum* was transmissible from New World monkeys to humans by mosquitoes and transferred back to monkeys via infected blood^16^, and New World monkeys could be experimentally infected with *P. malariae* from a human^27^. These findings suggested that the New World monkey parasite arose as an anthroponosis from *P. malariae*-infected humans^28^. However, comparisons of genetic diversities led some authors to conclude that the opposite scenario was also possible, i.e., that *P. malariae* originated from *P. brasilianum*^29–31^.

To resolve the number of *P. malariae*-related species and their host ranges, we used limiting dilution PCR to generate new sequences from faecal and blood samples of wild-living and captive chimpanzees and gorillas. We also developed a new approach to identify *P. malariae*-related sequences in published genome databases generated from apes with mixed *Plasmodium* infections. Here, we show that the clade of *P. malariae*-related parasites comprises three distinct lineages, one of which represents a previously unknown, distantly related species that infects wild-living chimpanzees, bonobos and gorillas across central and west Africa. We also show that “*P. malariae*-like”^24^ parasites represent a second ape-infective species, which is much more closely related to the human-infective lineage, but lacks evidence of genetic exchange with the human parasite. Moreover, comparison of genetic polymorphisms between the two lineages indicates that the human parasite has undergone a severe genetic bottleneck, followed by rapid population expansion. Finally, we show that *P. brasilianum* does not represent a distinct lineage but falls within the radiation of human *P. malariae* strains. These results thus place the origin of the *P. malariae*-related clade in Africa and indicate that the human parasite arose following a host switch of an ape parasite, while *P. brasilianum* arose after the transmission of the human parasite to New World monkeys.

## Results

### Three distinct lineages within the *P. malariae*-related group

Although wild apes have long been known to harbour *P. malariae*-related parasites^17^ (defined as a group of parasites that have much greater sequence similarity to *P. malariae* than to any other *Plasmodium* species, and which includes *P. brasilianum* and the human parasite itself, see Supplementary Note 1), only a few infections have been characterised. Previous field studies of wild apes and their mosquitoes demonstrated the presence of *P. malariae*-related parasites^9,21–23,32^, but recovered a very limited number of sequences, derived almost entirely from a single mitochondrial gene (*cytB*). To increase the number of parasite sequences for phylogenetic studies, we thus selected ape blood and faecal samples previously shown by diagnostic PCR to be *P. malariae*-positive, and subjected these to single genome amplification (SGA) targeting different genomic regions. SGA amplifies single templates and thus avoids generating parasite sequences with *Taq* polymerase artefacts, including chimeras produced by recombination, which is important for samples with multi-species infections. Using this approach we were able to amplify additional mtDNA and nuclear gene (*ldh* and *asl*) sequences. While most of these were derived from blood samples of sanctuary chimpanzees, *P. malariae*-related sequences were also recovered from one chimpanzee and two gorillas sampled in the wild (Supplementary Tables 1 and 2). We also derived newly assembled mtDNA genomes for the two “*P. malariae*-like” chimpanzee parasites GA01 and GA02.

**Fig. 1:**
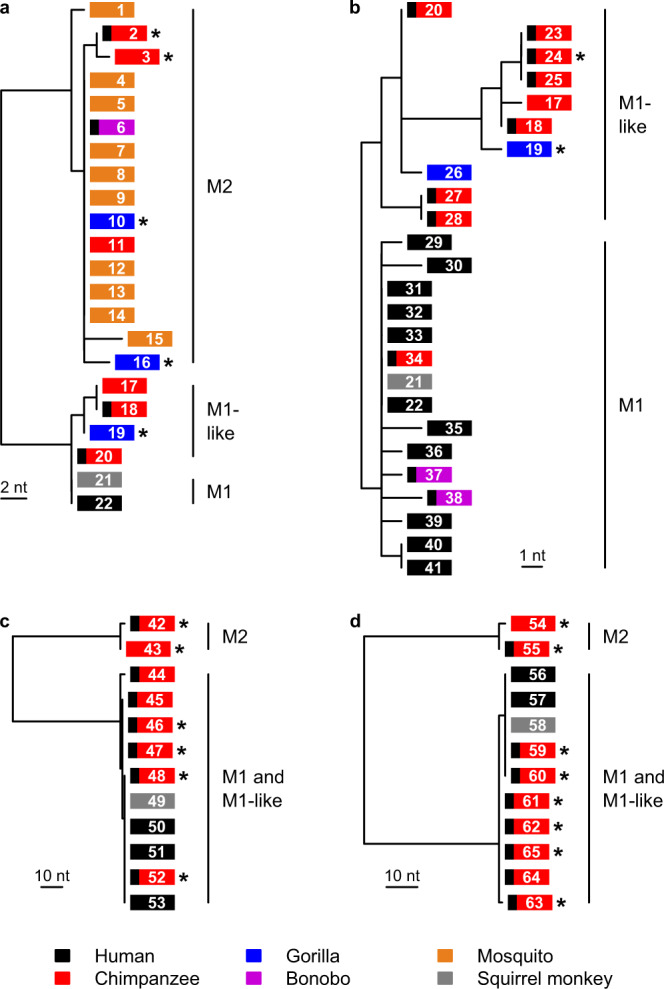
Phylogenetic relationships among M1 strains (including *P. malariae*) and the two ape-infective lineages M1-like and M2. **a** Maximum likelihood tree derived from a 641 bp mitochondrial DNA *cytB* fragment; this tree includes all available M2 *cytB* sequences, but for clarity includes only a few, representative M1 and M1-like sequences. **b** Maximum likelihood tree derived from a 2,038 bp mitochondrial *cytB*/*cox1* fragment; this tree includes all available sequences of sufficient length from these lineages, and was generated from a longer alignment that encompasses the region in part **a** (see Supplementary Fig. 1). **c** Maximum likelihood tree derived from a 682 bp *asl* fragment. **d** Maximum likelihood tree derived from a 724 bp *ldh* fragment. Boxes represent sequences and are coloured according to the host from which the sample was derived, as indicated in the key. A black bar at the left of a box indicates a sample from a captive or sanctuary ape; asterisks denote newly derived sequences; numbers in the boxes give the sequence code (Supplementary Table 2). Bootstrap support from 1,000 replicates was 100% for the nodes separating M2 from M1 and M1-like, and for the node separating M1 from M1-like in part **b**. Scale bars indicate the number of nucleotide changes.

Phylogenetic analyses of both newly derived (*N* = 21) and previously published (*N* = 44) parasite sequences revealed that *P. malariae* and primate parasites related to *P. malariae* fall into two highly divergent lineages in trees derived from both mtDNA (Fig. 1a, Supplementary Fig. 1) and nuclear (Fig. 1c, d) gene sequences. One of these (initially termed M1) included all *P. malariae* parasites from humans, together with *P. brasilianum* and some samples from apes. A second lineage (termed M2, see Supplementary Note 1) was quite distantly related to *P. malariae*, although clearly closer to *P. malariae* than to any other previously described *Plasmodium* species (see below). The M2 lineage included sequences obtained from samples from gorillas, chimpanzees and bonobos, as well as all 10 *P. malariae*-related sequences previously obtained from mosquitoes that feed on apes^23^ (Fig. 1a).

Closer examination of M1 sequences indicated that they could be divided into two lineages. In a tree derived from our longest available mtDNA alignment (~2 kb) for which we had numerous samples (Fig. 1b, Supplementary Fig. 1, Supplementary Tables 2 and 3), we could discern one clade comprising all human *P. malariae* sequences, *P. brasilianum* and a few sequences from captive apes, within which sequences differed by no more than two nucleotides from the consensus *P. malariae* sequence; we retained the term M1 for this clade (see Supplementary Note 1). The other non-M2 sequences showed greater diversity, and included samples derived from both wild and captive apes, including the two chimpanzee-derived strains (“*P. malariae*-like” GA01 and GA02) for which genome sequences had been derived^24^; here, we term these M1-like, to avoid confusion with the term “*P. malariae*-like” in the literature^23,24^ (see Supplementary Note 1). While M1 and M1-like strains were not separated in trees derived from partial nuclear gene sequences (Fig. 1c, d), subsequent comparisons of nuclear genome sequences confirmed that these two lineages are distinct.

### M2 represents a new *P. malariae*-related species that infects apes across Africa

**Fig. 2:**
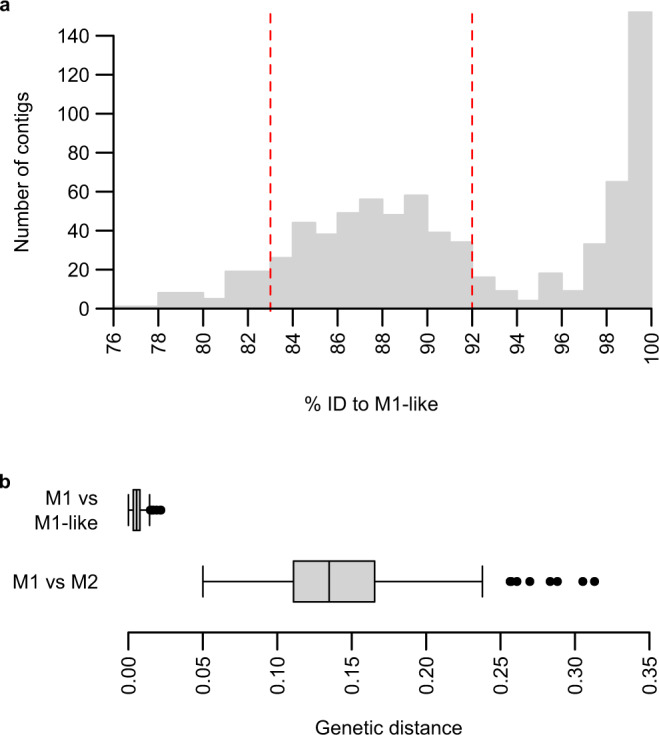
*P. malariae*-related contigs from chimpanzee sample PGABG03. **a** Identity to M1-like genome assembly PmlGA01 of de novo contigs assembled from PGABG03 sequencing reads mapped to *P. malariae*. To improve readability, one contig with a nucleotide identity of 70% has been omitted from the plot. Contigs between 83% and 92% nucleotide identity to PmlGA01 (dashed red lines) were assumed to originate from M2 and used for further assembly; contigs between 92% and 97% identity or below 83% identity were evaluated manually and included as M2 contigs if appropriate. **b** Boxplot of nucleotide divergences between M1 and M1-like, and between M1 and M2; *n* = 285 genes, totalling 350 kb of aligned sequence. A vertical centre line indicates the median; the box indicates the interquartile range; whiskers indicate 1.5x the interquartile range; black circles represent outliers.

To further characterise the M2 lineage, we first examined one blood sample from a sanctuary chimpanzee (SYptt92, sequences 2 and 55 in Fig. 1) that had yielded partial mitochondrial and nuclear sequences that clustered within M2. We attempted selective whole genome amplification (SWGA^11,33^) of whole blood DNA, but failed to obtain a sufficient number of sequences. As an alternative approach, we asked whether sequencing projects targeting any other ape *Plasmodium* species may have amplified sequences from M2 parasites as a by-product; we reasoned this was possible because M1 and M2 have previously been observed as coinfections with other *Plasmodium* species^9,24^. We thus screened parasite sequence databases from published *Laverania*^13^ (13 samples) and *P. vivax*-like^15^ (seven samples) infections of chimpanzees and gorillas for the presence of *P. malariae*-related sequences. We mapped all reads to a combined *Plasmodium* reference sequence including the *P. malariae* reference genome PmUG01, followed by de novo contig assembly of read pairs that mapped to *P. malariae* and alignment of contigs with the M1-like genome assembly PmlGA01^24^ (“*P. malariae*-like”). This approach identified one sample (PGABG03^13^) from a chimpanzee in a Gabonese wildlife reserve that yielded contigs with a bimodal distribution of nucleotide identities to the M1-like reference (Fig. 2a). One peak comprised contigs exhibiting more than 97% nucleotide identity to PmlGA01, while a second, broader peak was centred around 88% identity, suggesting coinfection with both M1-like and M2 parasites. We aligned high-quality contigs from this second peak (see Methods for details of how these were selected) with the *P. malariae* reference genome and annotated genes by automatic transfer followed by manual curation. Because the preparation of read libraries from the PGABG03 blood sample involved a whole genome amplification step^13^, DNA fragments may have been generated that were mosaics of coinfecting M1-like and M2 parasite genomes. We therefore performed several quality control steps to identify and remove mosaic contigs and other possible errors (see Methods for details). These assembly steps generated a partial (497 kb) genome sequence, with orthologues of 290 *P. malariae* genes annotated; however, most gene sequences were incomplete, and there were only 26 full-length genes. Although neither of the two nuclear genes amplified by SGA was covered entirely in this partial genome sequence, we identified 11 reads mapping to one of them (*asl*), yielding a consensus sequence with only two differences from one of the M2 amplicons (Fig. 1b, Supplementary Fig. 2). Although this *asl* sequence must be treated with caution due to the low read coverage, this similarity indicates that the divergent *P. malariae*-related genome obtained from the PGABG03 read library does indeed belong to the M2 lineage.

**Fig. 3:**
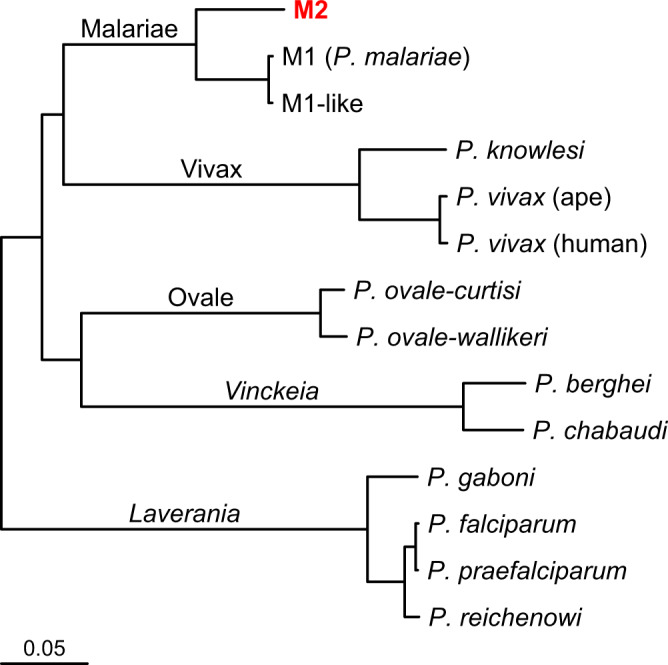
Relationship of the novel *P. malariae*-related species M2 to other mammalian *Plasmodium* species, including M1 (represented by *P. malariae*) and its close relative M1-like, from African apes. A maximum-likelihood tree was derived from protein alignments. Nucleotide sequences for 186 genes with one-to-one orthologues in all 14 genomes were translated and aligned, then gene alignments were cleaned and concatenated to a single alignment from which the tree was constructed. The five major lineages are labelled; for the two corresponding to subgenera the names are italicised. The tree has been rooted on the branch to the *Laverania*^36^. The new, divergent ape-derived lineage M2 appears at the top of the tree in red. Bootstrap support from 1,000 replicates was at least 99% for all nodes except that separating the Malariae and Vivax lineages from the rest of the tree. The scale bar represents 0.05 substitutions/site.

To characterise the relationship between the novel *Plasmodium* species and other malaria parasites, we aligned 285 genes annotated in M2 with their M1 and M1-like orthologues, using sequences from the PmUG01 assembly and sequences derived by single nucleotide polymorphism (SNP) calling from M1-like strain GA01^24^. We calculated corrected nucleotide divergences for individual genes (Fig. 2b, Supplementary Fig. 3) and for all sequences combined, yielding a genetic distance between M1 and M2 of 13.5%, compared with 0.6% between M1 and M1-like. Next, we generated amino acid alignments for 186 genes for which orthologues could be identified from mammalian parasite species from across the *Plasmodium* genus, calculated amino acid divergences between pairs of related parasites (Table 1) and constructed a protein tree (Fig. 3). This analysis confirmed that M2 clusters with the other *P. malariae*-related lineages M1 and M1-like, but is only distantly related to them. Moreover, the amino acid divergence between M1 and M2 (8.5%) was comparable to the divergence within the *P. vivax* lineage (9.0% between *P. vivax* and *P. knowlesi*), and greater than that within other mammalian *Plasmodium* lineages (Table 1). Such high divergence indicates that M2 reflects an ancient split within the *P. malariae*-related lineage, and should thus be considered a distinct species.

**Table 1:** Amino acid divergences between pairs of *Plasmodium* taxa.

Species 1	Species 2	% Amino acid divergence^a^	Relative divergence^b^
M1 (*P. malariae*)	M1-like	0.44	**1**
M1 (*P. malariae*)	M2	8.49	19.2
M1-like	M2	8.47	19.2
*P. falciparum*	*P. praefalciparum*	0.28	0.6
*P. falciparum*	*P. reichenowi*	1.58	3.6
*P. falciparum*	*P. gaboni*	5.37	12.2
*P. ovale-curtisi*	*P. ovale-wallikeri*	2.80	6.3
human *P. vivax*	ape *P. vivax*^c^	0.81	1.8
*P. vivax*	*P. knowlesi*	9.00	20.4
*P. berghei*	*P. chabaudi*	6.50	14.7

^a^From 186 protein sequence alignments.

^b^Divergence of each species pair relative to the divergence between M1 and M1-like.

^c^As yet it is unclear whether human *P. vivax* and ape *P. vivax* are separate species.

### M1 and M1-like parasites represent two non-recombining lineages

Analyses of mtDNA sequences (Fig. 1b) suggested a separation between, on the one hand, *P. malariae* sequences from humans (M1), and, on the other hand, *P. malariae*-related sequences obtained from wild apes (M1-like), although sequences from captive apes fell into both M1 and M1-like clades. This distinction was not apparent in comparisons of nuclear gene sequences (Fig. 1c, d), which showed less variability than the mtDNA sequences. Understanding the relationship between these two lineages is key to elucidating the zoonotic potential of parasites from wild apes, and so we attempted to increase the sampling of M1-like genomes. We performed SWGA^11,33^ on two *P. malariae*-positive samples, one from a Cameroonian sanctuary chimpanzee (MOpte51017) and the other from a wild-living chimpanzee (Ptv_Leo) from Cote d’Ivoire who had died from an infectious disease. We specifically selected these samples because they lacked other *Plasmodium* species and could thus be amplified without the risk of generating inter-species recombinants. Each sample was amplified on multiple occasions using different SWGA primer sets, with individual SWGA reactions pooled prior to MiSeq sequencing. Reads from MOpte51017 and Ptv_Leo were mapped to the *P. malariae* reference genome, covering 23.6 Mb and 0.3 Mb, respectively (Supplementary Table 4). For comparison, the two published M1-like genomes covered 20.4 Mb and 6.2 Mb of the same reference (Supplementary Table 4).

SGA sequences of *asl* and *ldh* from MOpte51017 were identical to those from *P. malariae* (sequence 52 in Fig. 1c, and sequence 60 in Fig. 1d), suggesting that this captive chimpanzee was infected with a human parasite, although an M1-like strain particularly closely related to M1 could not be excluded on the basis of these two short sequences. While the Ptv_Leo sample was obtained from a wild ape, the previously sequenced *cytB* amplicon was insufficient to determine whether this parasite represented an M1 or an M1-like infection^22,32^. To better characterise these samples, we combined the newly derived genome sequences with data from published human-derived M1 (i.e., *P. malariae*) parasites^24,34,35^ (*N* = 22) and chimpanzee-derived M1-like parasites^24^ (*N* = 2), identifying SNPs by comparison with the *P. malariae* reference genome, which we also included in the analysis. The genome sequences varied in quality, and in their coverage of the *P. malariae* reference (Supplementary Table 4), with very little of the reference genome covered by all strains.

**Fig. 4:**
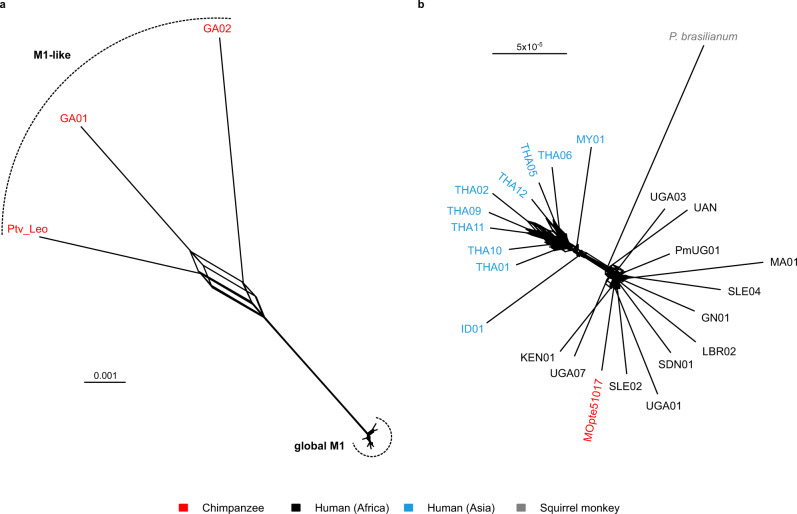
Relationships among M1 and M1-like strains. **a** Character-based phylogenetic network showing relationships between all three M1-like strains and a subset of the highest coverage M1 genomes (PmUG01, GN01, MA01, MOpte51017, MY01, UAN, THA05), based on 990 SNPs from 45 kb of sequence callable in all genomes. Dashed arcs indicate the M1 and M1-like groups of parasites. The reticulation at the centre of the network represents alternative paths introduced by incompatible SNPs, for example in which Ptv_Leo and GA02 share an allele rather than Ptv_Leo and GA01 as in most SNPs. **b** Character-based phylogenetic network of all available M1 genomes, including *P. brasilianum* and a sample derived from a chimpanzee (MOpte51017), based on 2,050 SNPs from 1.1 Mb of sequence. The two clusters correspond to strains from Asia (upper left cluster) and Africa (lower right cluster). *P. brasilianum* and MOpte51017 cluster with the African human strains. Scale bars indicate substitutions/site. Strain names are coloured according to their host or geographical origin, as indicated in the key.

A phylogenetic network generated from SNPs in genome regions covered by all four chimpanzee-derived parasite genomes and by the six highest coverage human-derived parasite genomes revealed a monophyletic M1 lineage that included MOpte51017 (Fig. 4a), confirming that the captive chimpanzee was indeed infected by a parasite of human origin. In contrast, the Ptv_Leo sequence was similar to the two previously characterised M1-like genomes (GA01 and GA02) in being quite distinct from the M1 clade, consistent with it being a wild-acquired chimpanzee parasite. These three M1-like genomes, plus the M1 clade, were all approximately equidistant (Fig. 4a), suggesting that the ancestor of the M1 clade was a member of the M1-like lineage.

Reticulation at the centre of the network (Fig. 4a) was consistent with gene flow among the various M1-like lineages, but there was little to indicate that there had been any exchange between M1 and any M1-like strain subsequent to the radiation within the M1 clade. Principal component analysis (PCA) of polymorphisms across 1.5 Mb of sequence across high-coverage M1 (*N* = 16) and M1-like (*N* = 2) strains was consistent with this conclusion, showing no M1 genomes intermediate between M1 and M1-like (Supplementary Fig. 4). We also searched for any genomic regions exhibiting unusually high diversity within M1, where some strains might have low divergence from M1-like, potentially reflecting recent introgression from an M1-like strain. Comparing each sample to the *P. malariae* reference genome, we plotted divergent SNPs per callable site for each strain in sliding windows along each chromosome; unlike the phylogenetic network and PCA, this approach allowed inclusion of genomic regions with coverage from only one or two strains.

**Fig. 5:**
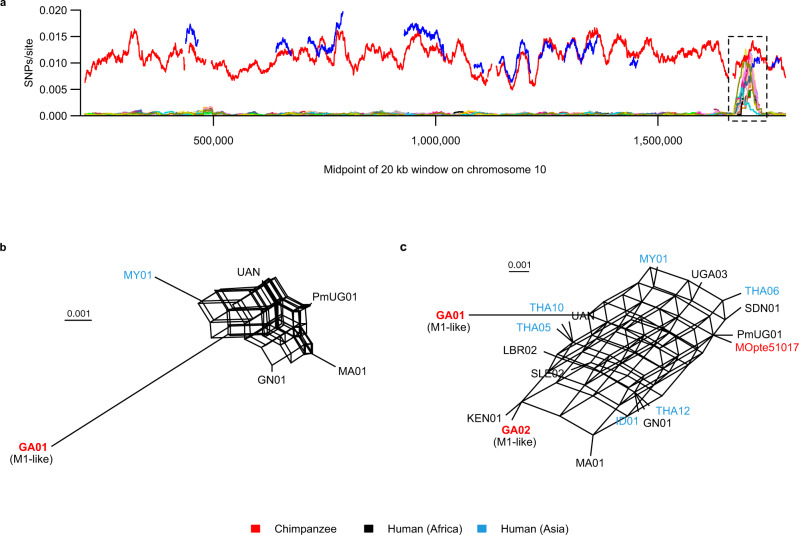
A short region of high M1 diversity on chromosome 10. **a** Divergence (SNPs per callable site) from the M1 reference genome (PmUG01) is plotted for windows of 20 kb with a 100 bp step size; values are plotted at the midpoint of each window. Subtelomeric regions were not analysed and are not shown in the plot, and low-complexity and uncallable sites were changed to N. Only windows with at least 40% non-N bases were included, leading to gaps in the lines. The M1-like strains are represented as thick red (GA01) and blue (GA02) lines, and the remaining thin coloured lines represent M1 strains. M1-like strain Ptv_Leo does not appear because it had no 20 kb windows on this chromosome that met the criteria for analysis. The dashed black box highlights a region of approximately 45 kb (positions 1,680,100-1,723,901) where many M1 strains have unusually high divergence from the reference. A more detailed view of this region is shown in Supplementary Fig. 6. **b** Character-based phylogenetic network generated from 415 SNPs in the high-diversity region, analysing M1-like strain GA01 and the five M1 strains that had coverage of most of the region. Pairwise nucleotide diversity among these M1 strains was 0.0063 substitutions per site. **c** Character-based phylogenetic network generated from 26 SNPs in the high-diversity region, analysing the 16 M1 and two M1-like strains with the highest genome coverage (Supplementary Table 4). To be included in the networks, we required a variant site to be covered by all strains, and many strains had coverage gaps at different sites, hence adding more strains usually decreased the number of SNPs analysed. Strain names in the networks are coloured according to their host or geographical orgin, as indicated in the key. Scale bars indicate 0.001 substitutions/site.

Across almost all of the genome (>99% of 20 kb windows in 22 Mb examined), each M1 strain differed from the reference at less than 0.001 SNPs per site, whereas the three M1-like strains (GA01, GA02 and Ptv_Leo) differed at ~0.005–0.015 SNPs per site (Supplementary Fig. 5). We found only one candidate region, extending over about 45 kb on chromosome 10, where some M1 strains were as divergent from the reference as GA01 was, while GA02 exhibited regions where divergence from the reference was unusually low (Fig. 5a, Supplementary Fig. 6). Across this region, no single strain was consistently divergent from the reference or consistently similar to GA02 (Supplementary Fig. 6). Thus, if this region does reflect introgression from an M1-like strain, it appears to have subsequently undergone recombination with other M1 strains. Consistent with this, phylogenetic networks derived from this region showed almost complete reticulation, whether based on only the small number of strains that included most of the region (Fig. 5b) or on more strains covering fewer SNPs (Fig. 5c, Supplementary Fig. 7). The region of high diversity extends across 12 genes, of which four appear to be house-keeping (the likely products are a translation initiation factor, ADP-ribosylation factor, methionine-tRNA ligase, and U2 snRNA/tRNA pseudouridine synthase), while two encode proteins of unknown function conserved in *P. falciparum* and *P. vivax*, and the other six are unidentified and have no clear orthologues in other *Plasmodium* species. It is therefore unclear how or why the novel alleles might have been selectively retained following introgression. These findings suggest that, in this region of chromosome 10, variation among human-derived M1 strains reflects hybridisation with an M1-like strain from apes, but since this region covers only about 0.2% of the genome sequence examined, introgression appears to have been very rare.

### Reduced diversity and increased nonsynonymous polymorphism in M1

Previous studies comparing human *Plasmodium* species (i.e., *P. falciparum* and *P. vivax*) with their close relatives infecting apes have revealed different levels and patterns of nucleotide polymorphism in parasites from the two hosts^11,12,36^. For *P. malariae*, the phylogenetic network (Fig. 4a) indicated that the M1-like lineage had considerably higher diversity than M1, consistent with a previous study of a smaller dataset^24^. Calculating mean pairwise nucleotide diversity (π), we found that genetic variation among the three M1-like parasites from chimpanzees was 1.04%, nearly 40-fold higher than the value (0.026%) seen for M1 strains (Table 2). To investigate the possibility that the higher diversity among M1-like strains was caused by an elevated rate of sequencing errors, we compared the numbers of changes that were due to transition or transversion mutations, restricting the analysis to sites within coding sequences where all possible changes are synonymous (fourfold degenerate sites). Sequencing errors are much more often transitions than transversions^37^, and so a data set with more errors is expected to have a higher transition:transversion ratio than one with fewer errors. However, the transition:transversion ratio for SNPs in M1-like (1.25) was slightly lower than that in M1 (1.31), indicating that increased sequencing error is unlikely to explain the much greater diversity in M1-like.

**Table 2:** Nucleotide diversity and polymorphisms in M1 and M1-like parasites.

Statistic^a^	M1 (*P. malariae*)^b^	M1-like^b^
*π*	0.00026	0.01043
*π*_0_	0.00024	0.00761
*π*_4_	0.00040	0.02241
*π*_0_/*π*_4_	0.594	0.340
NS	1981	13,073
S	1031	10,594
NS/S	1.921	1.234
Neutrality index	1.551	0.996

^a^*π*, mean pairwise nucleotide diversity; *π*_0_, mean pairwise diversity at zerofold degenerate sites; *π*_4_, mean pairwise diversity at fourfold degenerate sites; NS, number of nonsynonymous polymorphisms; S, number of synonymous polymorphisms.

^b^Divergence between M1 and M1-like is 0.00963 substitutions/site.

The difference in pairwise nucleotide diversity between M1 and M1-like strains was about 32-fold at sites where all changes result in a change to the amino acid sequence (zerofold degenerate sites), but about 56-fold at fourfold degenerate sites (Table 2), indicating that M1 has a relative excess of nonsynonymous diversity compared with M1-like. Calculating the ratio of nonsynonymous to synonymous polymorphisms for each lineage, we found that while both lineages had more nonsynonymous than synonymous polymorphisms, the ratio (NS/S) was considerably higher for M1 (1.92) than for M1-like (1.23). Among fixed differences between M1 and M1-like strains, the NS/S ratio was 1.24, yielding a neutrality index^38^ (NI; calculated by dividing the ratio for polymorphisms by that for fixed differences) of 0.996, very close to the neutral expectation of 1. In contrast, the NI for M1 was 1.551, again indicating an excess of nonsynonymous polymorphisms, and suggesting that it is the M1 lineage that is unusual in this regard.

**Fig. 6:**
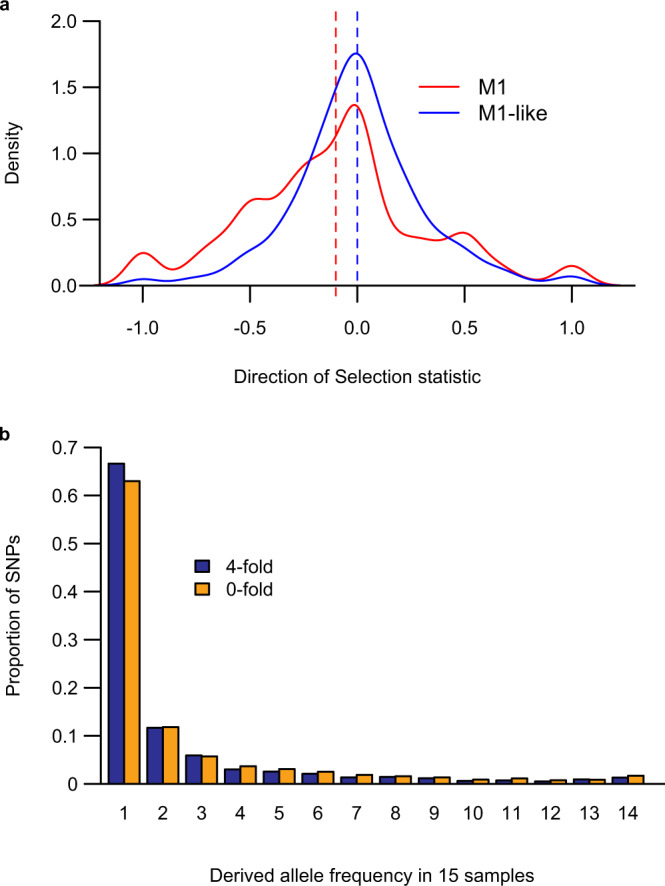
Synonymous and nonsynonymous nucleotide polymorphisms in M1 and M1-like. **a** Direction of Selection (DoS) values for M1 and M1-like. The density plots show the distribution of the DoS statistic in M1 (red) and M1-like (blue) for 903 genes with defined values of DoS in both lineages. Vertical dashed lines indicate the median DoS value for each lineage. **b** Unfolded site frequency spectra of SNPs at 1102 fourfold (blue) and 4583 zerofold (yellow) degenerate sites in M1.

We wanted to examine the distribution of this pattern of nonsynonymous polymorphism across genes. Many genes in M1 had no synonymous polymorphisms, and thus the NI value could not be defined. Instead we used the Direction of Selection (DoS) statistic^39^, which contrasts the fraction of nonsynonymous changes among fixed differences with that among polymorphisms, and can be calculated for genes with no synonymous polymorphisms. DoS values for M1-like strains were centred around the neutral expectation of 0, whereas for M1 strains the entire distribution was shifted towards negative values (Fig. 6a), indicating that a higher fraction of polymorphisms than of fixed differences were nonsynonymous. Thus, the excess of nonsynonymous polymorphisms in M1 is not due to a small number of unusual genes but is common across the genome.

The site frequency spectrum (SFS) of polymorphisms is expected to vary dependent on the level of selection, with more strongly deleterious mutations expected to be rarer. Synonymous changes are usually assumed to be neutral, and so the SFS of polymorphisms at fourfold degenerate sites can be used as an approximation of the neutral expectation. The unfolded SFS for polymorphisms at zerofold degenerate sites in M1 strains did not show the expected excess of these nonsynonymous changes in the singleton class, compared to polymorphisms at fourfold degenerate sites (Fig. 6b). This result suggests that these nonsynonymous polymorphisms in the M1 lineage have been subject to little or no purifying selection.

### Origin of *P. brasilianum*

In our analyses of mtDNA and nuclear loci, the *P. brasilianum* sequence was identical to that of *P. malariae* from humans (Fig. 1). Previous studies considering the origin of *P. brasilianum* have similarly used data from only a small number of loci^4,24–26,29–31^. To investigate this question in more detail, we compared the *P. brasilianum* genome^40^ to numerous *P. malariae* sequences, generating a phylogenetic network from 2,050 SNPs covered in all M1 strains (Fig. 4b). This network divided human M1 (i.e., *P. malariae*) into two clusters, corresponding to strains from Africa and Asia, as reported recently^35^. Unsurprisingly, the M1 sample from a captive Cameroonian chimpanzee (MOpte51017) clustered with African strains of *P. malariae*. Addition of the M1-like strain GA01 to this network suggested that the M1 root lay near the base of the African cluster (Supplementary Fig. 8), although resolution in this region is poor because the network is dominated by differences between M1 and M1-like. *P. brasilianum* clustered with the African strains of *P. malariae* (Fig. 4b), suggesting that its origin was a human strain imported to South America from Africa.

## Discussion

It has been known for about a century that chimpanzees are infected by parasites morphologically indistinguishable from *P. malariae* in humans^41,42^. The chimpanzee parasite was named *P. rodhaini*^17^, but the question of whether the ape and human parasites represent two distinct species was debated, with the eventual consensus being that they do not^16^. Here we show that *P. malariae*-related sequences from ape samples in fact belong to two quite divergent species, and that both infect gorillas as well as chimpanzees. We expect that these two species are morphologically similar (i.e., cryptic species) and that both may have been present in the original microscopic studies. One of the ape parasite species is very closely related to *P. malariae*, but there is little evidence of recent genetic exchange between it and the human parasite. These findings, together with observations on the patterns of genetic diversity in ape and human parasites, have allowed us to finally untangle the origins of *P. malariae*, as well as the origins of *P. brasilianum* in New World monkeys.

Molecular characterisation of the ape parasite species more closely related to *P. malariae* began with mtDNA sequences a little over 10 years ago^32,43,44^ and was extended to near-complete nuclear genomes by Rutledge et al.^24^ in 2017, who termed these parasites *P. malariae*-like (we have used M1-like above). Rutledge and colleagues clearly considered *P. malariae* and *P. malariae*-like to represent two separate species, and indeed suggested that the two may have split several million years ago^24^. However, with the characterisation of a third *P. malariae*-like genome, our analyses indicate a different scenario: *P. malariae* falls within the radiation of the *P. malariae*-like parasites, being no more divergent from the ape parasites than they are from each other (Fig. 4a). Moreover, the fact that the human parasites exhibit substantially less genetic variation than the strains from apes (Fig. 4; Table 2) indicates that *P. malariae* arose from a recent transmission of an ape parasite to humans. *P. malariae* has clearly experienced a severe genetic bottleneck, which is most parsimoniously attributed to the cross-species transmission event. The increased fraction of polymorphisms within *P. malariae* that are nonsynonymous, compared to polymorphisms within *P. malariae*-like, is consistent with rapid population expansion subsequent to that bottleneck. Our scan across the core genome found only a single region, on chromosome 10, where diversity among *P. malariae* strains is substantially higher than elsewhere in the genome. This region amounts to less than 0.21% of the core genome, suggesting that there has been very little introgression from ape parasites into the human parasite population since it started to diversify following its zoonotic origin.

This relationship between the human and ape *P. malariae*-related parasites is remarkably similar to that between human *P. falciparum* and ape parasites from the subgenus *Laverania. P. falciparum* appears to have been derived from one species of gorilla parasite (*P. praefalciparum*) quite recently, exhibits much reduced genetic diversity relative to the gorilla parasites, and there is little sign of introgression from the gorilla parasites subsequent to the origin of the human parasite^13^. No evidence has been found of *P. praefalciparum*, or other *Laverania*, infecting humans living in regions close to infected apes^22,45,46^, just as *P. malariae*-related infections of ape origin have not been found in humans^22^.

A notable difference between the two *Plasmodium* clades concerns their host specificity. In the wild, ape *Laverania* appear to be strongly host-specific: the three species infecting gorillas have not been found in wild chimpanzees, while the four species infecting chimpanzees and/or bonobos (two extremely closely related hosts) have never been detected in samples from wild gorillas^36^. The basis of these barriers to cross-species transmission of *Laverania* are, to some extent, understood^47^. In contrast, the two *P. malariae*-related ape parasite species have been found in samples from both chimpanzees and gorillas, and there seems no reason a priori to expect that they could not infect humans. Indeed, some 70–80 years ago, it was reported that *P. malariae* could be transmitted to chimpanzees^48^, and that “*P. rodhaini*” (the *P. malariae*-related parasite seen in chimpanzee blood) could be transmitted to humans^49^. However, most of these experiments involved inoculation of parasitised blood, where the number of parasites transmitted would greatly exceed those from a mosquito bite. Later, Bray was able to infect *Anopheles gambiae* mosquitoes with *P. malariae* from humans, which were then able to transmit these parasites to chimpanzees^20^. These observations are consistent with our finding of *P. malariae* of human origin infecting captive apes. In contrast, Bray was unable to infect *A. gambiae* with *P. malariae*-related parasites from chimpanzees, and he concluded that the risk of a zoonotic transmission of *P. malariae*-related parasites from chimpanzees to humans was much lower than the reverse anthroponosis. Indeed, our genomic analyses indicating a scarcity of introgression from ape parasites into *P. malariae* provide direct evidence that zoonotic transmission is extremely rare, and that *P. malariae* has effectively become a new species, isolated from the related ape parasites. If recognised as such, *P. malariae*-like might retain the name *P. rodhaini* or, given that the original “*P. rodhaini*” was likely a mix of two divergent species, it could be renamed as *P. praemalariae*, recognising its role in the origin of the human parasites. Importantly, until we know the basis of the apparent isolation between the ape and human parasites, we cannot exclude the possibility that *P. praemalariae* could emerge in humans again.

It has long been known that there is a very close relationship between human *P. malariae* and *P. brasilianum*, a parasite infecting New World monkeys, but the direction of transfer between hosts has been debated, even quite recently^31^. Now that *P. malariae* has been shown to have a relatively recent African origin, it is clear that *P. brasilianum* arose subsequent to the export of the human parasite to South America, most likely within the last 500 years. As yet, only a single *P. brasilianum* genome has been characterised^40^, and so it remains unknown whether these parasites, which have been found to infect around 30 different monkey species in several countries across South and Central America^50^, are the result of a single introduction, or multiple transmissions, from humans.

The existence of the second *P. malariae*-related ape parasite, termed M2 above, was first suggested by an analysis of mtDNA sequences (Fig. 1a; see also ref. ^36^). We have assembled a partial M2 genome sequence, from reads that had gone unnoticed in a multiply-infected chimpanzee sample used to generate a *P. gaboni* genome sequence^13^. These data indicate that the extent of divergence between M2 and *P. malariae* is similar to that between *P. vivax* and its relatives infecting Asian monkeys (e.g., *P. knowlesi*), and greater than that among the most divergent *Laverania* species, such as *P. falciparum* and *P. gaboni* (Fig. 3; Table 1). Thus, M2 parasites clearly represent a previously undescribed species, for which we suggest the name *Plasmodium celatum* (in reference to its being hitherto hidden). Sequences from this parasite have been found in samples from chimpanzees, bonobos and western gorillas, as well as in mosquitoes, with the latter species, *A. vinckei*, found to be infected with a variety of other chimpanzee and gorilla *Plasmodium* parasites^23^. Moreover, the M2-positive samples were collected at several sites in Cameroon, Gabon and the Republic of the Congo, as well as one site in the eastern Democratic Republic of the Congo. Thus, this parasite is widely distributed and appears to exhibit no host specificity in apes, although it has never been found in humans. Since two of the three species within the *P. malariae*-related parasite lineage have only been found infecting African apes, while the third reflects a recent zoonosis, it seems clear that this lineage evolved in Africa, and indeed it seems highly likely that the entire radiation of mammalian *Plasmodium* parasites originated there^36^.

The timescale of the origins of the *Plasmodium* species infecting humans has been the subject of much speculation. Rutledge and colleagues estimated that the divergence of *P. malariae* and *P. malariae*-like occurred at the same time as the divergence, within the *Laverania*, of the ancestors of *P. falciparum* and the chimpanzee parasite *P. reichenowi*^24^. They suggested that these two events in the two distinct lineages each involved a move between non-human primates and humans, and that their similar timing may indicate that they both resulted from a common underlying historical event^24^. However, it is now clear that neither of these divergence events involved ape-to-human transmissions. Indeed, it was already known that this divergence within the *Laverania* involved the split between the ancestors of *P. reichenowi* and *P. praefalciparum*, a gorilla parasite^9,51^, and that the gorilla-to-human transmission that gave rise to *P. falciparum* occurred much later^11,13^. Similarly, our analyses now indicate that the common ancestor of *P. malariae* and any one genome from *P. malariae*-like, as analysed by Rutledge et al., in fact reflects the coalescence of alleles within *P. malariae*-like, and again the ape-to-human transfer giving rise to *P. malariae* occurred much later. One estimate suggests that *P. falciparum* originated about 50,000 years ago^13,43^, although we have suggested a more recent timeline, of 5000–10,000 years ago^36^. Whatever the age of *P. falciparum*, the origin of *P. malariae* is similarly recent: levels of genetic diversity in *P. malariae* are even lower than in *P. falciparum*^36^, although this may be due in part to a slower mutation rate in the quartan parasite^24^. Thus, there may indeed have been a common historical event that promoted the emergence of both *P. falciparum* and *P. malariae* in humans, such as the beginnings of agriculture in Africa.

## Methods

### Ape DNA samples

Ape samples were selected from existing specimen banks previously collected from captive and wild-living chimpanzees and gorillas in west and central Africa (Supplementary Table 1). Whole blood and dried blood spots were obtained from central (*Pan troglodytes troglodytes)* and Nigeria-Cameroon (*P. t. ellioti*) chimpanzees housed at the Sanaga Yong (SY) Chimpanzee Rescue Center and the Mfou (MO) National Park Wildlife Rescue Center in Cameroon^10,12^. Additionally, a small amount of blood (SAggg3157) was obtained from a western lowland gorilla (*Gorilla gorilla gorilla*), who was killed by hunters and confiscated by the anti-poaching programme of the Cameroonian Ministry of Environment and Forestry^12^. Spleen and lung samples were collected from a wild-living habituated western chimpanzee (*P. t. verus*) (Leo), who died in 2002 in the Tai Forest in Cote d’Ivoire^32^. Finally, faecal samples were collected non-invasively from non-habituated apes at numerous sites across equatorial Africa^9,10,21,52^. DNA was extracted from whole blood and dried blood spots using the QIAamp Blood DNA Mini Kit or the Puregene Core Blood Kit (Qiagen). Faecal DNA was extracted using the QIAamp Stool DNA mini kit (Qiagen). All specimens were subjected to host mtDNA analysis to confirm their species and subspecies origin. Sample collection at the time was approved by the Ministry of Environment and Forestry of the respective countries, and all samples were shipped in compliance with Convention on International Trade in Endangered Species of Wild Fauna and Flora regulations and country-specific import and export permits.

### Diagnostic PCR for *P. malariae-*related parasites

Ape blood and faecal samples were screened for *P. malariae*-related sequences by diagnostic PCR using both pan-*Plasmodium* and *P. malariae-*specific primer sets as previously described^9,21^. Briefly, a 956 bp mitochondrial *cytB* fragment was amplified using pan-*Plasmodium* primers, and a 600 bp mitochondrial *cytB* fragment was amplified using *P. malariae*-specific primers (Supplementary Table 5). Amplicons were sequenced without interim cloning and subjected to phylogenetic analysis to determine the species of the amplified *Plasmodium* sequences. The chimpanzee spleen sample Ptv_Leo was previously reported to be positive for *P. malariae*^32^.

### Limiting dilution PCR

Ape samples were subjected to SGA to derive *Plasmodium* sequences without PCR-induced artefacts^9,10,12,21,52^. Sample DNA was endpoint diluted in 96-well plates, and the dilution that yielded less than 30% positive wells was used to generate single template derived sequences. For *P. malariae-*positive samples, mitochondrial (*cytB* and 2.5 kb *cox1/cytB*), and nuclear (*ldh, asl*) gene regions were amplified using *P. malariae*-specific primer sets (Supplementary Table 5, Supplementary Fig. 1) and previously reported amplification conditions^9,10,21^.

### Selective whole genome amplification

Near full-length M1 and partial M1-like genomes (respectively) were amplified from chimpanzee blood (MOpte51017) and spleen (Ptv_Leo) samples using SWGA as previously described^11,12^. Briefly, custom scripts were used to identify sequence motifs (6–12 bp) that occurred frequently in the *P. malariae* reference genome^24^, but only rarely in the human genome. These were filtered to remove primers that exhibited extreme melting temperatures, were predicted to form homodimers, or bound the *P. malariae* mitochondrial genome or its subtelomeric regions more than three times. The resulting primer sets, Pm_set37 and Pm_set31 (Supplementary Table 6), exhibited high frequency of occurrence in the *P. malariae* genome and low frequency of occurrence in the human genome. SWGA was performed by amplifying whole blood or tissue DNA (100–750 ng) in a 50 μl reaction with 1× phi29 buffer, 1 mM dNTPs, 3.5 μM of SWGA primers (an equimolar mix of primers in the set), 1% BSA and 30 units of phi29 polymerase (New England Biolabs). SWGA conditions included a 1 h ramp-down step (35–30 °C), followed by an amplification step for 16 h at 30 °C, followed by a phi29 denaturation step for 10 min at 65 °C. SWGA products were purified using AMPure Beads (Beckman Coulter) and stored at 4 °C. To obtain sufficient quantities of parasite genomic DNA for sequencing, ape-derived DNA samples were subjected to multiple rounds (up to 4) of successive SWGA amplification, some of which were performed with alternating primer sets to improve genome coverage. Pooled SWGA products were MiSeq sequenced and the MiSeq reads trimmed using cutadapt^53^ to remove adaptor sequence.

### Data mining and assembly of an M2 genome

Full details of the bioinformatics methods used in this section and the next are given in Supplementary Note 2. Briefly, published read libraries^13,15^ were filtered to remove host reads by mapping to the host reference genome using bwa^54^ then mapped to a combined *Plasmodium* reference with bwa followed by a more stringent mapping with smalt (www.sanger.ac.uk/science/tools/smalt-0; -y 0.6, i.e., 60% of bases required to be identical to the reference). Read pairs with both mates mapped to the *P. malariae* reference genome PmUG01 were considered “*P. malariae*-related” reads and assembled de novo with SPades^55^. De novo contigs of at least 500 bp with k-mer coverage of at least 2X were aligned to the M1-like genome assembly PmlGA01^24^ using MUMmer^56^, and contigs that were not mapped were aligned to PmUG01. Additionally, each *P. malariae*-related read set was aligned to existing M2 sequences for *asl* and *ldh* using smalt (-y 0.9, i.e., 90% of bases required to be identical to the reference), generating one *asl* sequence (number 42) used in Fig. 1c.

Sequence derived from sample PGABG03 (ERS333073^13^) was used to generate the M2 assembly. M2 contigs were identified by similarity to PmlGA01 or PmUG01: contigs with 83–92% nucleotide identity to either reference were assumed to be M2, and contigs that had less than 83% or 92–97% nucleotide identity were inspected manually to evaluate if they were likely to be M2-derived. Pseudochromosome scaffolds were generated from the M2 contigs by ordering against PmUG01^24^ using ABACAS^57^. To reduce assembly errors near contig ends, 100 bp were removed from the end of each contig, followed by re-extension with IMAGE^58^. Two checks were performed to identify misassemblies, including misincorporation of M1-like sequence: an alignment of *P. malariae*-related reads to the initial M2 assembly was inspected to identify contigs for which multiple different haplotypes were represented in the mapped reads; and blastn alignments of the assembly to PmUG01 and PmlGA01 were inspected to identify contigs with segments that had unusually high identity to PmlGA01 or that mapped to different regions of the reference genomes.

Coding sequences in the final M2 assembly were annotated by transfer from PmUG01 using RATT^59^, followed by manual inspection and curation. Corrected genetic distances were calculated in R^60^ (TN93 model) from nucleotide alignments of each gene from M2 with its orthologues from PmUG01 and M1-like strain GA01. Amino acid alignments were generated for genes that had one-to-one orthologues in all *Plasmodium* species in the analysis (Fig. 3) and used to find the number of differences between pairs of species and thus calculate inter-species distances.

### Comparison of M1 and M1-like strains

Chimpanzee-derived read libraries (Ptv_Leo, MOpte51017, GA01 and GA02^24^) were filtered to remove sequencing reads from the host and from coinfections with other *Plasmodium* species by mapping with bwa to a combined chimpanzee and *Plasmodium* reference. Read pairs aligned to *P. malariae* were used for variant calling, in combination with human-derived M1 read libraries from three recent studies^24,34,35^. Variants from the reference (PmUG01) were called and filtered using GATK^61^ with HaplotypeCaller, excluding subtelomeres (Supplementary Table 7), low-complexity regions (identified using Dustmasker^62^) and sites where an individual sample had more than one alternative allele. Where a heterozygous SNP call was made, the sample genotype was taken to be the allele supported by most reads. Coverage of the reference genome for each genome (Supplementary Table 4) was calculated with GATK using CallableLoci, considering sites covered by at least five reads to be callable. SNPs in the *P. brasilianum* genome^40^ were identified using MUMmer^56^ by aligning assembled contigs with the *P. malariae* reference and identifying differences, since raw sequencing data were not available for this isolate.

Principal components were calculated for variants in the highest coverage M1 and M1-like genomes (Supplementary Table 4) using plink^63^. Unfolded M1 site frequency spectra were obtained for sites that were fourfold or zerofold degenerate in PmUG01 using est-sfs unfolder^64^, with M1-like strain GA01 as the outgroup. To identify regions of high diversity or divergence, SNPs/callable base was calculated for each M1 and M1-like strain in sliding windows of 20 kb in the reference genome with a 100 bp step size, excluding windows with fewer than 8,000 callable bases.

To examine polymorphism, gene sequences were generated for each strain by changing the reference sequence to the alternative allele at variant sites, or to N at uncallable sites, and then discarding individual gene sequences with low coverage of the reference. The remaining sequences, including PmUG01, were used to generate nucleotide alignments for non-subtelomeric, non-pseudogenes. Synonymous and nonsynonymous polymorphisms and fixed differences were counted by determining the effect of the change on PmUG01 or GA01. To calculate mean pairwise nucleotide diversities, positions that were uncallable in any strain were excluded, and diversity was calculated in R^60^ for all remaining sites and for sites that were fourfold and zerofold degenerate in PmUG01.

### Phylogenetic analyses

Nucleotide sequences were aligned with Clustal Omega^65^ (Fig. 1) or TranslatorX/MUSCLE^66^ (all other analyses) and trimmed to include only sites present in all samples. Amino acid sequences were aligned with MUSCLE^67^ and cleaned with Gblocks^68^. Phylogenetic trees were constructed with RAxML^69^ algorithm a, with 1000 bootstrap replicates, using either the GTR-Γ model (for nucleotide alignments) or PROTGAMMAJTTF model (for amino acid alignments). Phylogenetic networks were generated by SplitsTree4^70^ with the MedianNetwork transform, using variant sites identified by SNP calling and excluding sites that were non-biallelic or were uncallable in the subset of strains used for each network. To convert the number of changes into changes per site, the amount of sequence callable in all strains in the sequence set was calculated from the CallableLoci output bed files using bedtools^71^ merge and genomecov.

### Reporting summary

Further information on research design is available in the Nature Research Reporting Summary linked to this article.

## Supplementary information

Supplementary Information

Reporting Summary

## Data Availability

The new sequence data reported in this paper have been deposited in the appropriate NCBI databases under accession numbers MN175636-MN175639, MZ555468-MZ555486, MZ927539 (GenBank, SGA sequences) and PRJNA767638 (Sequence Read Archive, SWGA sequences), as noted in Supplementary Table 1. Scaffolds from the new M2 assembly derived from sequence library ERS333073 are available in the Third Party Annotation Section of the DDBJ/ENA/GenBank databases under the accession numbers TPA: BK061131-BK061144. The study also analysed publicly available data from BioProjects PRJEB14392^24^ and PRJNA344798^40^ (genome assemblies), and BioProjects PRJEB13344^24^, PRJEB12680^34^ and PRJEB37746^35^ (read libraries), details in Supplementary Table 4.
