# Supplementary material for: Zoonotic origin of the human malaria parasite Plasmodium malariae from African apes
Source: Nat Commun. 2022 Apr 6;13:1868. doi: 10.1038/s41467-022-29306-4 (PMC8987028; doi:10.1038/s41467-022-29306-4)
Supplement: Supplementary file 1 — Supplementary Information [file 41467_2022_29306_MOESM1_ESM.pdf]

SUPPLEMENTARY INFORMATION

Supplementary Note 1. Nomenclature.

The nomenclature of parasites within the *P. malariae* lineage has evolved in recent years as more information about related primate parasites has been forthcoming. We use the term "*P. malariae*-related" to describe all parasites within this lineage that are more similar to *P. malariae* than to any other previously described human malaria parasite species. We use M1 to include *P. malariae* from humans, as well as near-identical strains from some captive African apes, and *P. brasilianum* from New World monkeys; here we establish that all of these parasites are of human origin. We use M1-like to include parasites closely related to *P. malariae* but identified in wild apes. Sequences from M1-like strains are distinct from M1, and exhibit more diversity than M1, especially when longer/larger sequences are compared. We also found M1-like sequences in some samples from captive apes. We use M2 to denote a highly divergent species of *P. malariae*-related parasites found in samples from chimpanzees, gorillas, bonobos and mosquitoes across central Africa. Previously, the term "*P. malariae*-like" has been used by Rutledge et al. (2017)[1], while the same authors also used "*P. malariae*-like 1" and "*P. malariae*-like 2" in Makanga et al. (2016)[2]. Our analyses find that "*P. malariae*-like" and "*P. malariae*-like 2" correspond to M1-like, while "*P. malariae*-like 1" corresponds to M2.

When first studied, *P. malariae*-related parasites from apes were termed *P. rodhaini*[3]. In retrospect it is unclear whether those parasites included strains of M1, and/or M1-like, and/or M2. We propose that M1 parasites from apes, as well as those from humans, should be referred to as *P. malariae*. The name *P. brasilianum* should be retained for M1 strains from New World monkeys, unless it is established that there is no reproductive barrier between *P. brasilianum* and *P. malariae*. As it is apparent that *P. malariae* was originally derived from M1-like parasites in apes, and yet M1 and M1-like appear to have become isolated from each other, we propose the name *P. praemalariae* for M1-like. For the previously unknown ("hidden") species M2, we propose the name *P. celatum.*

Supplementary Note 2. Detailed bioinformatic methods.

Data mining and assembly of M2 genome

Read libraries from samples sequenced by Otto *et al.*[4] and Gilabert *et al.*[5] were filtered to remove host reads by mapping to either the chimpanzee or gorilla reference genomes, using bwa[6,7] (version 0.7.12) with default settings. Unmapped read pairs were extracted with SAMtools[8] (version 1.9) and screened for *P. malariae*-related sequences by mapping with bwa to a combined *Plasmodium* reference consisting of genomes of *P. malariae*, *P. ovale-curtisi*[1], *P. adleri*, *P. billcollinsi*, *P. blacklocki*, *P. gaboni*, *P. praefalciparum*, *P. reichenowi*[4] and *P. vivax*[9]. Libraries were discarded unless at least 20,000 sequencing reads were in read pairs with both mates mapped to *P. malariae*. Retained samples were then filtered more stringently by mapping sequencing reads to the same reference with smalt (www.sanger.ac.uk/science/tools/smalt-0, version 0.6.7), requiring 60% of bases in each read to be identical to the reference (-y 0.6), and then discarding samples where more than 10% of mapped read pairs with at least one mate mapped to *P. malariae* had the other mate mapped to a different species. Read pairs with both mates mapped to *P. malariae* were extracted with samtools and considered "*P. malariae*-related" reads.

P. malariae-related reads for each read library were assembled using SPades[10] (version 3.14.1, using default settings including error correction). *De novo* contigs of at least 500 bp with k-mer coverage of at least 2X were aligned to the M1-like genome assembly PmlGA01[1] ("*P. malariae*-like") using MUMmer[11] (version 4.0.0beta2, contigs initially aligned with nucmer using -maxmatch and -l 12 options; filtered with delta-filter using default options; tiled with show-tiling using -i 40, -v 40, -V 1, -l 1 and -R options), and their distribution of nucleotide identity to the reference visualised in R. Additionally, each *P. malariae*-related read set was aligned to existing M2 sequences MBptt781_SGA4.3 (*asl*) and SYptt_SGA4.4 (*ldh*) using smalt with high-stringency settings (-y 0.9, i.e., 90% of bases required to be identical to the reference), and any reads mapped were used to generate a consensus sequence, although this yielded only one *asl* sequence (number 42 in Fig. 1c).

For sample PGABG03 (accession number ERS333073), which had a distribution of contig identities that suggested the presence of both M1-like and M2 coinfections, contigs with M1-like alignments of less 60% of their length were aligned to the *P. malariae* reference genome PmUG01[1] (M1) using the same settings. Contigs that had less than 60% of their length included in an alignment to one or other reference were discarded. Contigs that had at least 97% nucleotide identity to either reference were assumed to be M1-like, and those that had between 83 and 92% identity were assumed to be M2. Contigs with between 92 and 97% or less than 83% identity were inspected individually, and added to the M2 contig set if they had no apparent misassemblies, had a similar level of divergence from both M1 and M1-like references in blastn alignments[12] (version 2.9.0), and this divergence was greater than the divergence between M1 and M1-like in the same region. The M2 and M1-like contig sets were ordered against PmUG01 using ABACAS[13] to produce initial assemblies. To identify misassemblies and other errors, including M1-like/M2 chimeric contigs, the *P. malariae*-related reads were mapped back to a reference consisting of the initial M2 and M1-like assemblies and contigs that had not been included in either set, and reads mapping to M2 were visually inspected in Geneious Prime (version 20202.1.2., https://www.geneious.com) to identify contigs for which multiple different haplotypes were represented in the mapped reads. In addition, the initial M2 assembly was compared with the M1 and M1-like assemblies using blastn, and visually inspected using ACT[14] (version 18.0.2) to identify contigs that had segments aligned to PmUG01 with nucleotide identity more consistent with M1-like than M2 (>97%), and contigs that had segments that aligned to another region of the genome. Because manual inspection suggested that SPades had introduced some assembly errors at contig ends, 100 bp was trimmed off the end of each contig. Contigs were then re-extended where possible using IMAGE[15] (version 2.4), performing two iterations with a k-mer of 41 followed by two iterations with a k-mer of 31, and using sequencing reads that had aligned to the presumed M2 contigs or unassigned contigs. The misassembly checks were then repeated. In total, 18 contigs out of a original total of 494 were deleted following these checks, and four longer contigs were edited to

remove short regions of incorrect sequence, resulting in a final assembly of 497,491 bp. Annotations were transferred from the *P. malariae* reference genome to the final M2 assembly using RATT[16], followed by manual inspection and correction, which included adjusting annotations to exclude possible erroneous sequences at the ends of contigs, and resulted in the annotation of orthologues of 290 *P. malariae* genes. Most annotations were of incomplete fragments, with 26 representing full-length genes.

To address the possibility that the “M2” genes actually represented extremely divergent alleles from an M1-like parasite, from loci under strong diversifying selection, we used the “Gene Ontology Enrichment Analysis” tool in PlasmoDB[17] (release 55) to analyse the *P. malariae* orthologues of annotated M2 genes. This survey confirmed that there was no enrichment for genes with GO terms indicating that they are likely to be under diversifying selection.

M2 comparison

For individual gene alignments, genes annotated in M2 and their orthologues in PmUG01 (identified during gene annotation) were screened to exclude pseudogenes and low-complexity regions masked using segmasker[12], and alignments were generated for 285 genes using TranslatorX/MUSCLE[18,19] (versions 1.1 and 3.8.31, respectively). Sequences derived by variant calling from M1-like strain GA01 (as in the “Variant calling” section, except the Dustmasker step was skipped because the gene alignments had already been masked for low-complexity sequence) were added to these alignments, following the PmUG01 sequence alignment. Sites with missing data or alignment gaps were removed and corrected genetic distances were calculated for individual genes and for a concatenated alignment of all genes using the ape R package[20] (version 5.4.1, dist.dna, model “TN93”).

For comparison with other *Plasmodium* species, amino acid alignments were generated for orthologous genes from M2, *P. malariae*[1], M1-like (as above), *P. knowlesi*[21], human and ape *P. vivax*[9,22], *P. ovale-curtisi*, *P. ovale-wallikeri*[1], *P. berghei*, *P. chabaudi*[23], *P. falciparum*[24], *P. praefalciparum*, *P. gaboni*[5] and *P. reichenowi*[9]. Orthologue groups for the

published genomes were obtained from PlasmoDB[17], except for ape *P. vivax* and *P. ovale-wallikeri*, which were added to the groups based on their orthology to human *P. vivax* (taken from reference 22) and *P. ovale-curtisi* (using OrthoMCL[25], version 2), respectively. Orthologue groups with exactly one representative in each species assembly (1:1 orthologues) were used for analysis, 186 genes in total. For each gene, nucleotide sequences from each genome assembly were translated and the resulting amino acid sequences were aligned with MUSCLE[19]. Gene alignments were cleaned with Gblocks (version 0.91b) with default settings[26] and concatenated to produce a single alignment of 63,621 amino acids. Distances between pairs of species were calculated by counting the number of differences and dividing by the length of the sequence.

M1 and M1-like sequences

Host filtering of newly-derived (Ptv_Leo and MOpte51017) and published (GA01 and GA02[1]) sequencing libraries was performed by mapping with bwa to a combined reference containing both chimpanzee and *Plasmodium* reference genomes (chromosomes only, *P. malariae*, *P. ovale-curtisi*, *P. falciparum*, *P. vivax* and *P. gaboni*). Read pairs mapping to *P. malariae* (either both mapped to *P. malariae* or one read unmapped) were extracted and used for SNP calling. The sequencing libraries from samples GA01 and GA02 were kindly provided by the study authors[1] to allow the use of the complete, unfiltered read sets. For M1, we included genome data from patients sampled in three recent studies[1,27,28] and assembly contigs for *P. brasilianum*[29]. Subsequent analyses revealed two pairs of genomes from one study[28] that were near-identical within the pair; only the genome from each pair with greater coverage of the reference (KEN01 and THA02) was included in the final analysis.

To obtain mitochondrial DNA sequence from GA01 and GA02 read libraries, reads were mapped to the chimpanzee genome with bwa, then non-chimpanzee reads were mapped with smalt to a combined reference of mtDNA genomes from *P. falciparum* (AY282930[30]), *P. malariae* (LT594637[1]), *P. ovale-curtisi* (HQ712052[31]) and *P. vivax* (LT635627[9]). All read pairs where either mate mapped to any genome in this reference were extracted and used

for de novo contig assembly with SPades. Blastn to the NCBI Genbank database was used to identify *P. malariae*-related sequence from the resulting contigs.

Variant calling

Variants in M1 and M1-like genomes were called using the Genome Analysis Toolkit[32] (GATK, version 4.1.6.0 except where noted). Sequencing reads were mapped to a combined reference of *P. malariae* (PmUG01), *P. falciparum*, *P. ovale-curtisi* and *P. vivax* (chromosomes only) with bwa, and PCR duplicate reads removed. De-duplicated bam files were used to generate genomic variant call format files for *P. malariae* chromosomes using HaplotypeCaller (with OverClippedReadFilter option). Variants were called jointly on all samples, and single nucleotide polymorphisms (SNPs) were filtered using a set of annotations and values that were optimised to exclude subtelomeric SNPs (assumed to be mainly artefactual) while retaining as many SNPs as possible at fourfold degenerate sites (assumed to be mainly correct) (Quality <30.0, QualByDepth <2.0, RMSMappingQuality <42.0, FisherStrand >60.0, StrandOddsRatio >3.0, MappingQualityRankSumTest <-2.0 or >4.0, ReadPosRankSumTest <-8.0 or >8.0). The ability to call a SNP was assessed for each site in the reference for each sample using CallableLoci (GATK version 3.8.1.0, minimum read depth 5, OverClippedReadFilter applied), which output a bed format file annotating callable sites. To simplify downstream analyses, chromosome sequences were then generated for each sample by changing the reference sequence to the alternative allele at variant sites using bcftools[33]. Low-complexity regions in the reference were identified using Dustmasker[12] (version 1.0.0), and bases were changed to N in a given sample if they were in low-complexity regions or subtelomeres, or in that sample were uncallable, had more than one alternative allele or had allele depth <5. For biallelic sites with heterozygous SNP calls, the allele supported by the most reads was used (or one allele was selected at random if read support was equal for both). At the start and end of each chromosome, the boundary of the core genome defined by Ibrahim *et al*.[28] was compared with the extent of the region in which the PmUG01 (M1) and PmIGA01 (M1-like) genomes were syntenic, and whichever of

these was closer to the centre of the chromosome was used as the core/subtelomere boundary (Supplementary Table 7).

Raw sequencing reads from *P. brasilianum*[29] were not available, so instead contigs from the published assembly were aligned with PmUG01 using MUMmer[11] (contigs aligned with nucmer with default options, filtered with delta-filter, then final alignments produced using show-tiling with -1, -i 90, -u 50 and -l 1000 options), followed by identification of single-nucleotide differences using show-snps (-C option). This information was then used to generate new chromosome sequences by changing the reference sequence to the alternative allele at variant sites, a step which allowed this sample to be included in the analysis pipeline used for the other samples. Regions of the reference that had been covered by contig alignments were identified with show-coords. Regions that were not covered in the alignments, low-complexity regions and subtelomeres were changed to N. Inspection of the *P. brasilianum* gene sequences aligned with other M1 strains identified clusters of differences from the reference genome, frequently close to the ends of *P. brasilianum* contigs. These differences may represent genuine sequence changes but could also have arisen due to sequencing or assembly errors, and without access to the sequencing reads it is difficult to validate or dismiss them. Because the differences were usually unique to *P. brasilianum*, we reasoned that if artefactual they would be unlikely to influence the qualitative relationship between *P. brasilianum* and other M1 strains, but could incorrectly inflate estimates of M1 diversity. We therefore included *P. brasilianum* in the M1 network but excluded it from diversity and polymorphism analyses.

Genome comparisons

When chromosome sequences had been generated for all strains, these were concatenated with each other and the reference sequence to produce a sequence alignment for each chromosome, then converted to a haploid vcf file using snp-sites[34]. For each analysis, appropriate sample subsets were extracted from the vcf files using bcftools[33] (version 1.9), removing sites that were missing data, invariant or non-biallelic in the sample subset.

Principal components were calculated from haploid vcf files of high-coverage M1 and M1-like samples (Supplementary Table 4) using plink[35] (version 1.9).

To search for regions of unusually high diversity in M1 or low divergence between M1 and M1-like, sliding windows of 20 kb with a 100 bp step size were examined for each sample. For each window, SNPs/base was calculated by determining the number of differences between that sample and the reference using the haploid vcf files and determining the number of non-N bases from the chromosome alignments, excluding windows with fewer than 8,000 non-N bases. Only windows completely contained in the chromosome core were analysed. For increased resolution around the high-diversity region reported in the text, a plot was also generated for chromosome 10 using windows of 5 kb with a 100 bp step size and a minimum of 2,000 non-N bases.

Site frequency spectra were generated from a haploid vcf file for all M1 strains except *P. brasilianum* (N=23). Since many SNPs had data missing from at least one genome, leading to variation in the number of calls, the number of calls at each SNP position were down-sampled to 15, a value chosen as a compromise between increasing genome count and increasing SNP count. Fourfold and zerofold degenerate sites were identified in PmUG01. Outgroup alignments were generated from PmUG01 and SNP-derived GA01 sequences and used with the est-sfs unfolder[36] (version 2.03) to identify the derived allele at each position and calculate the unfolded site frequency spectrum.

Polymorphism analysis

Nucleotide alignments of orthologues were generated for each non-subtelomeric, non-pseudogene from PmUG01, using reference genes that had been masked for low-complexity sequence as above. Additional M1 strains were excluded if less than 80% of the reference length was unmasked. All M1-like strains with at least some sequence unmasked were included for every gene, unless all three strains had some sequence, in which case only strains with at least 50% of the reference length unmasked were included. For both M1 and M1-like, strains were excluded if they contained an internal stop codon. Synonymous

and non-synonymous polymorphisms were counted by determining the effect of a polymorphism on the M1 reference PmUG01 or the M1-like strain GA01, considering only genes and sites with at least two strains for both M1 and M1-like. Synonymous (S) and non-synonymous (NS) fixed differences were counted by comparing codons between PmUG01 and GA01, excluding sites that were polymorphic in either M1 or M1-like, and assuming the order of changes that gave the lowest number of NS changes. The neutrality index[37] (NI) was arrived at by calculating the total NS and S over all genes for polymorphisms and for fixed differences, and dividing NS/S for polymorphisms by NS/S for fixed differences. The Direction of Selection statistic[38] (DoS) was calculated for each gene by subtracting the fraction of polymorphisms that were NS from the fraction of fixed differences that were NS, and density plots were generated using the "density" function in R with a fixed bandwidth of 0.075 for both M1 and M1-like. For calculation of pairwise diversities, positions that were masked in any sequence were excluded from the alignments, after which genes with less than 100 bp remaining in the alignment were discarded. Mean pairwise nucleotide diversities were calculated at all remaining sites and at sites that were fourfold and zerofold degenerate in PmUG01 using the ape R package[20] (dist.dna, model "raw").

Characterisation of a putative region of introgression

Looking for evidence of introgression between M1 and M1-like parasites, we identified one candidate region, extending over about 45 kb on chromosome 10, where some M1 strains were as divergent from M1-like parasites as they were from each other (Fig. 5a, Supplementary Fig. 6). This region is located towards one end of chromosome 10, approximately 495 kb before the telomere, and contains 12 genes. To investigate whether the extraordinarily high diversity among M1 strains could be explained by causes other than introgression from M1-like, we examined the identity of these genes. The first six genes have clear syntenic orthologues in both *P. falciparum* and *P. vivax*, including two with unidentified functions (PmUG01_10045700 and PmUG01_10046000). However, none of the *P. falciparum* or *P. vivax* orthologues show the same striking elevation of diversity that we

observed in *P. malariae* (data from Malariagen[39,40]). We queried PlasmoDB's OrthoMCL annotations[17,25] for related genes, and found that with one exception, these genes have no paralogues within *P. malariae*, but numerous homologues in other species. The exception is PmUG01_10046100, which is a methionine-tRNA ligase gene that is the result of an ancient *Plasmodium* duplication that gave rise to one gene encoding a protein targeted to the cytoplasm, and another encoding a protein targeted to the apicoplast. The second part of the high-diversity region has no discernible synteny with the corresponding regions in *P. falciparum* and *P. vivax* and we were unable to identify orthologues in other *Plasmodium* species of the six genes it contains. Using ExportPred[41] (as implemented in PlasmoDB[17]), we established that none are predicted to be exported. Of the six genes, five encode products described as "hypothetical proteins". One is annotated to encode a "conserved *Plasmodium* protein of unknown function", but we were unable to identify any homologues of this gene. Three genes have no paralogues in the *P. malariae* genome. The other three are apparently related to one another and to six other *P. malariae* genes, but also form part of a group of homologous genes (OG6_100908) with 344 members across numerous *Plasmodium* species, so seem unlikely to represent a novel *P. malariae* multigene family. In conclusion, we could find no reason why the genes in this region should be subject to unusual diversifying selection, nor other explanations for the exceptional pattern of diversity among M1 strains. Thus, the region has most likely resulted from introgression from an M1-like strain, but we can identify no reason why this is the only chromosomal region where diversity persists.

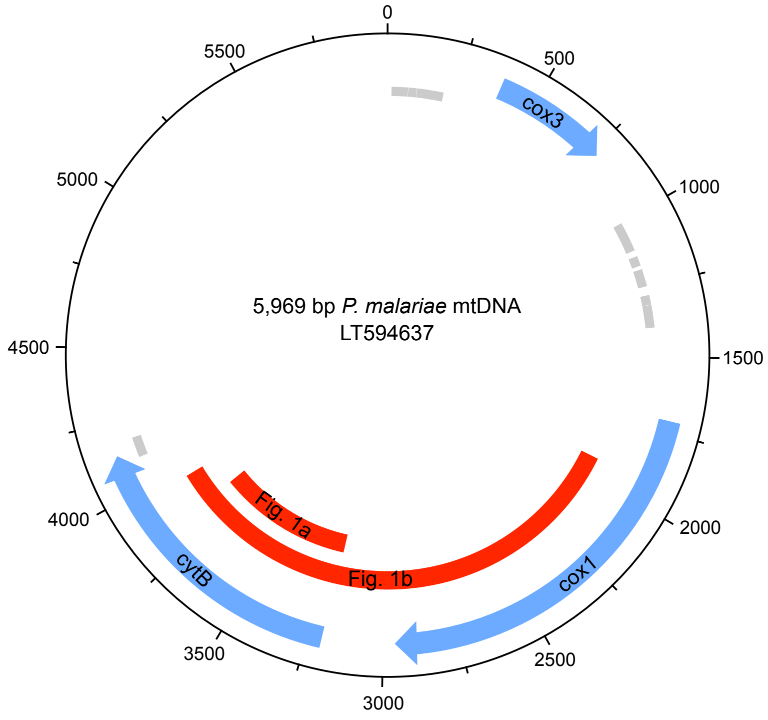

Supplementary Figure 1. Location in the *P. malariae* mitochondrial genome of the sequences used to generate the trees in Fig. 1a and 1b. The regions included in the alignments are indicated by red arcs, and shown relative to the positions of protein-coding genes (cytB, cytochrome B; cox1/3, cytochrome C oxidase subunit 1/3) in blue, and to rRNA gene sequences in grey. Base numbering is according to the mtDNA from the PmUG01 assembly (accession LT594637). The schematic was generated using DNAPlotter[42].

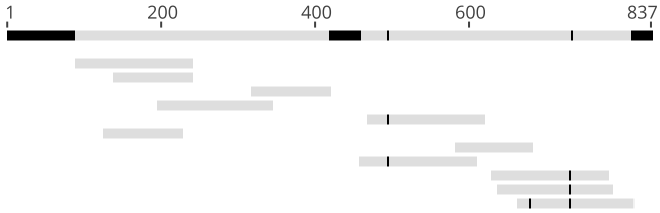

Supplementary Figure 2. *P. malariae*-related reads from sample PGABG03 mapped to M2 *asl*. Reads which had been filtered by mapping to PmUG01 were aligned to M2 sequence amplified by SGA from sample MBptt781 (top), using smalt with high stringency settings. Read alignments were visualised in Geneious, showing aligned reads as shorter grey boxes underneath the reference sequence. Vertical black bars indicate a difference between the read and reference; longer black regions in the reference had no read coverage. Altogether, the PGABG03 *asl* reads covered 682 bp of the 837 bp target, in two regions separated by a 40 bp gap that was spanned by two read pairs. The majority consensus sequence of these reads has been included in the tree shown in Fig. 1c (sequence number 42).

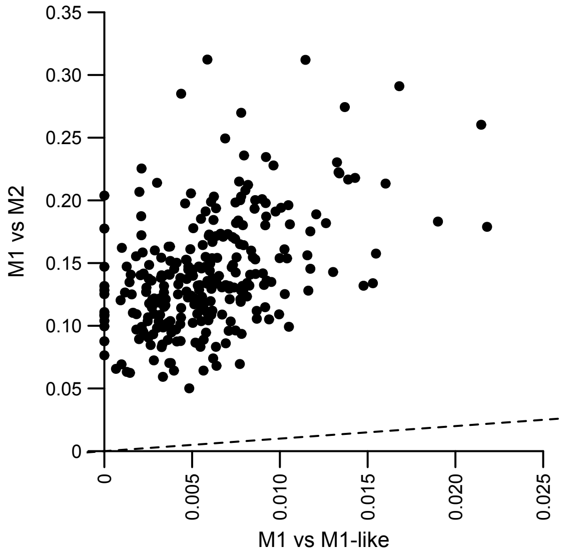

Supplementary Figure 3. Divergence in coding sequences between M1, M1-like and M2. The plot shows corrected genetic distance between M1 (PmUG01) and M2, plotted against corrected genetic distance between M1 (PmUG01) and M1-like (GA01), showing values for 273 genes that were annotated in the new M2 genome assembly and had alignments of at least 200 bp. The dashed line has a slope of 1.

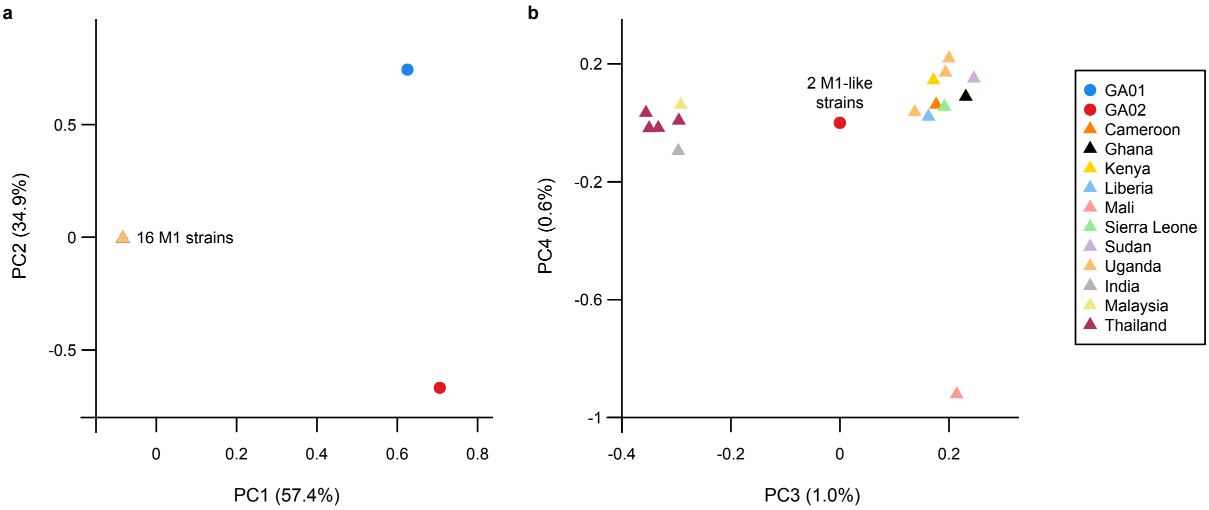

Supplementary Figure 4. Principal component analysis of variation among two M1-like strains (circles) and 16 M1 strains (triangles). The analysis includes PmUG01 and the 15 other M1 strains with the highest genome coverage (Supplementary Table 4), but excludes M1-like strain Ptv_Leo, which had low coverage; 1.5 Mb of the genome was analysed. **a** Position of strains on the first two principal components (PCs), which together explain 92.3% of the variance. **b** Position of strains on the third and fourth principal components, which together explain 1.6% of the variance. Colours denote the country of origin of M1 strains, or the identity of M1-like strains, as indicated in the legend.

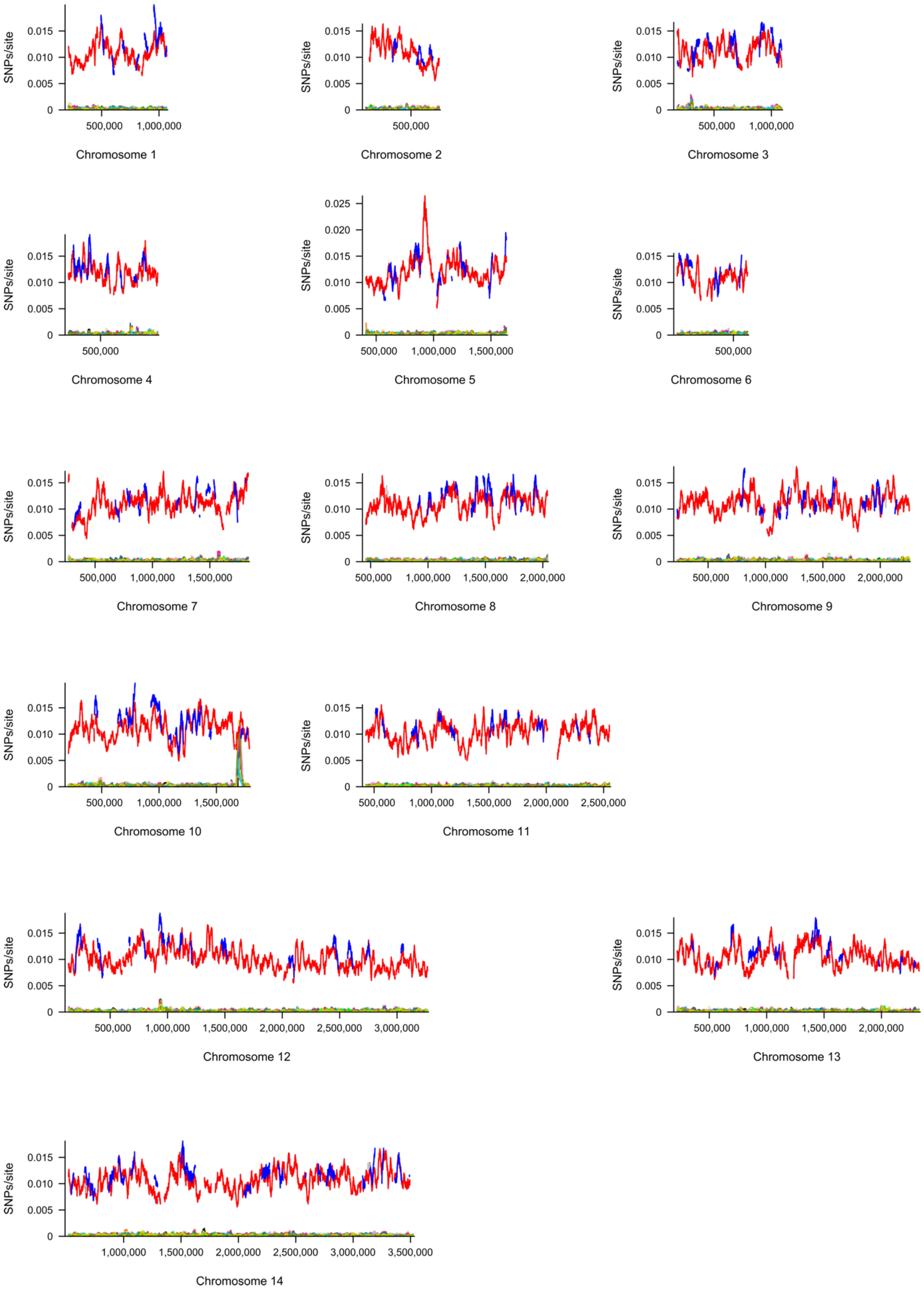
SNPs/site
0.015
0.010
0.005
0
500,000
1,000,000
Chromosome 1
Chromosome 2
Chromosome 3
Chromosome 4
0.025
0.020
1,500,000
Chromosome 5
Chromosome 6
Chromosome 7
2,000,000
Chromosome 8
Chromosome 9
Chromosome 10
2,500,000
Chromosome 11
3,000,000
Chromosome 12
Chromosome 13
3,500,000
Chromosome 14

Supplementary Figure 5. Genetic diversity along each chromosome. For each chromosome, divergence (SNPs per callable site) from the M1 reference genome (PmUG01) is plotted for windows of 20 kb with a 100 bp step size; values are plotted at the midpoint of each window. Only windows with at least 40% non-N bases were included, leading to gaps in the lines. Subtelomeric regions were not included, and low-complexity regions were masked. The M1-like strains are represented as red (GA01), blue (GA02) and grey (Ptv_Leo) lines; the remaining coloured lines represent M1 strains.

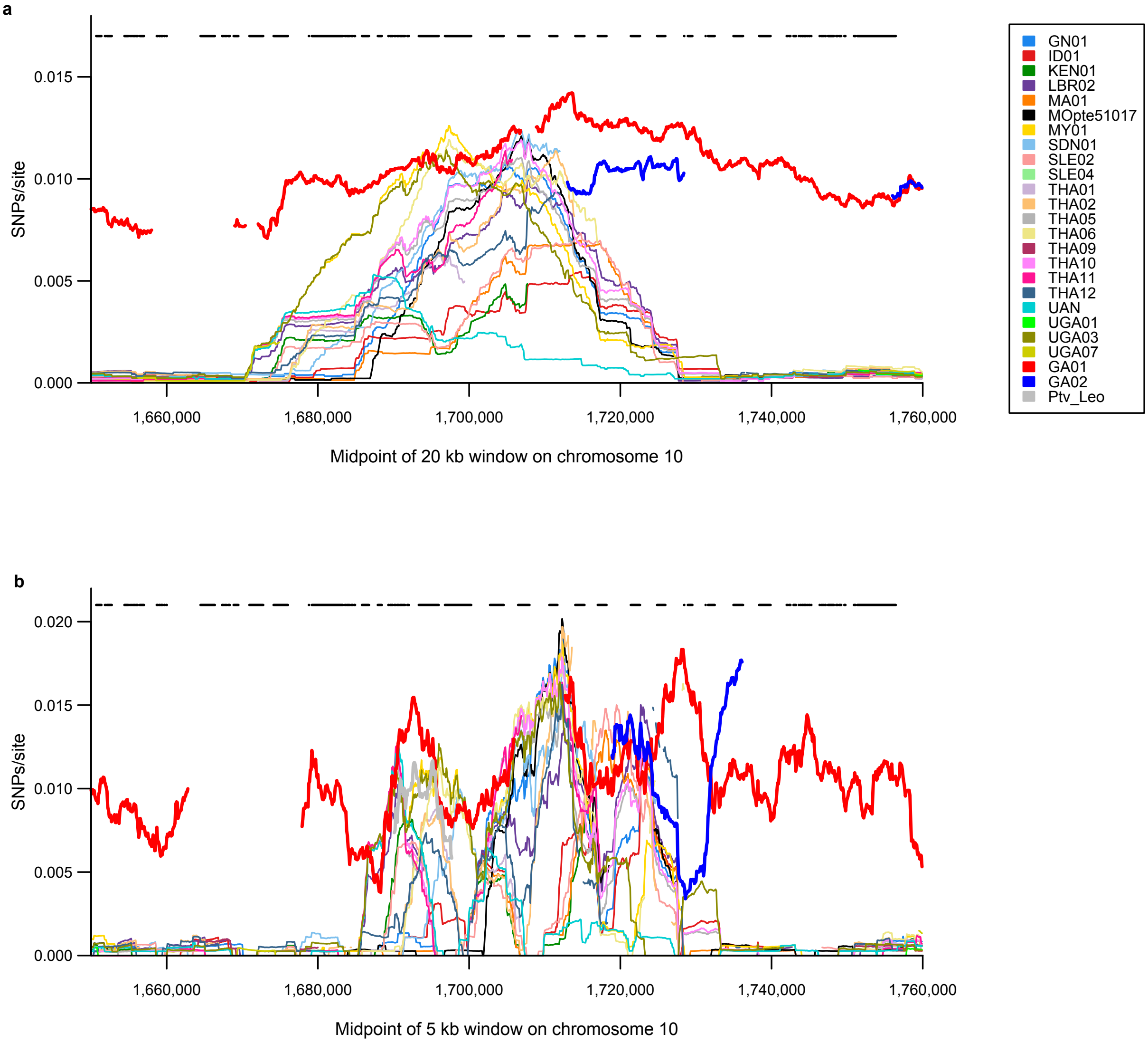

Supplementary Figure 6. Genetic diversity in a region of high M1 diversity on chromosome 10. Divergence (SNPs per callable site) from the M1 reference genome (PmUG01) is plotted as thick lines for M1-like strains GA01 (red), GA02 (blue) and Ptv_Leo (grey), and as thin lines for M1 strains (see colour code at top right). Only windows with at least 40% non-N bases were included, leading to gaps in the lines. Horizontal black lines at the top of the plot indicate the location of coding exons. **a** Windows of 20 kb, and step size of 100 bp. **b** Windows of 5 kb, and a step size of 100 bp.

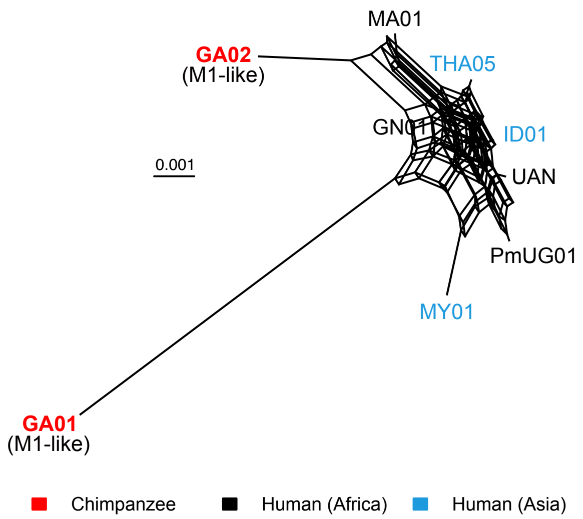

Supplementary Figure 7. Phylogenetic network of the region of high M1 diversity on chromosome 10. Character-based phylogenetic network generated from 144 SNPs in the high-diversity region (positions 1,680,100-1,723,901 on chromosome 10), analysing seven M1 and two M1-like strains. Compared with the networks in Fig. 5, this network is based on fewer SNPs than Fig. 5b but includes more M1 strains, and is based on more SNPs than Fig. 5c but includes fewer M1 strains. Strain names are coloured according to their host or geographical origin, as indicated in the key; the scale bar indicates 0.001 substitutions/site.

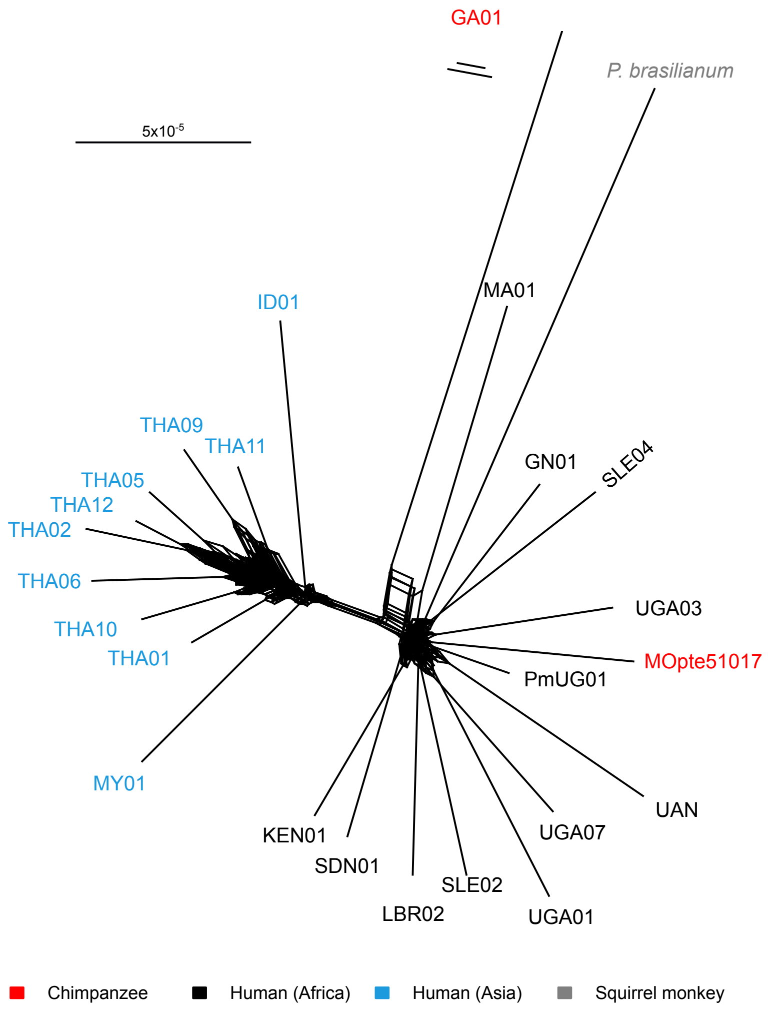

Supplementary Figure 8. Phylogenetic network of all M1 strains and M1-like strain GA01. The character network is based on 10,685 SNPs from 910 kb of sequence, of which 1,736 were polymorphic in M1. The branch to GA01, which has a length of 0.0098 substitutions/site, has been truncated to show the M1 network region more clearly. Strain names are coloured according to their host or geographical origin, as indicated in the key; the scale bar represents $5x10^{-5}$ substitutions/site.

Supplementary Table 1. Ape samples used to amplify *P. malariae*-related gene and genome sequences.

Sample ID	ID	Species	Sub-species	Collection date	Country	Location	Sample	*Plasmodium* spp. present[a]	Gene	Length (bp)	Sequence name	Reference	Accession no.
SYptt92	Lucy	chimpanzee	Ptt	08/10/2013	Cameroon	SY sanctuary	blood	Pg, M2	*cytB*	772	SYptt92_SGA3.1	this study	MN175636
									ldh	725	SYptt92_LDH_SGA4.4	this study	MZ555480
MBptt781	n/a	chimpanzee	Ptt	24/04/2005	Cameroon	Mambele	faecal	Pg, M2	*cox1/cytB*	2,531	MBptt781_FD_SGA2.1	this study	MN175639
									asl	837	MBptt781_ASL_SGA4.3	this study	MZ555470
									ldh	724	MBptt781_LDH_SGA2.9	this study	MZ555482
SAggg3157	n/a	western gorilla	Ggg	n/a	Cameroon	unknown	blood	Pb, Pp, Pv, M2	*cytB*	956	SAggg3157_SGA30.55	this study	MN175638
GTggg618	n/a	western gorilla	Ggg	04/04/2005	Republic of Congo	Goualougo Triangle	faecal	Pb, M2	*cytB*	746	GTggg618_SGA10.1	this study	MN175637
									cox1/cytB	2,529	GTggg618_FD_SGA1.2	this study	MZ555468
SYptt65	Xeko	chimpanzee	Ptt	08/02/2013	Cameroon	SY sanctuary	blood	Pg, M1-like	*cytB*	955	SYptt65_SGA20.10	43	MF693450
									cox1/cytB	2,529	SYptt65_FD_SGA1.1	this study	MZ555469
SYptt61	Simoosa	chimpanzee	Ptt	20/02/2013	Cameroon	SY sanctuary	blood	Pg, M1 or M1-like	*asl*	836	SYptt61_ASL_SGA1.1	this study	MZ555476
									ldh	724	SYptt61_LDH_SGA1.3	this study	MZ555483
SYptt63	Tic	chimpanzee	Ptt	26/02/2013	Cameroon	SY sanctuary	blood	Pg, Pr, M1-like	*cox1/cytB*	2529	SYptt63_SGA20.3	43	=SYpte66_SGA20.2
									ldh	724	SYptt63_LDH_SGA2.1	this study	=SYptt61_LDH_SGA1.3
SYpte66	Yoko	chimpanzee	Pte	12/02/2013	Cameroon	SY sanctuary	blood	Pg, M1-like	*cytB*	955	SYpte66_SGA20.8	43	MF693448
									cox1/cytB	2529	SYpte66_SGA20.2	43	MF693447
									asl	831	SYpte66_ASL_SGA1.1	this study	MZ555477
									ldh	724	SYpte66_LDH_SGA2.10	this study	MZ555484
											SYpte66_LDH_SGA1.1	this study	=SYptt61_LDH_SGA1.3
MOpte51016	n/a	chimpanzee	Pte	05/02/2007	Cameroon	Mfou sanctuary	dried blood spot	M1	*cytB*	2,497	MOpte51016_SGA20.1	43	MF693452
									asl	837	MOpte51016_ASL_SGA20.1	this study	MZ555478
									ldh	863	MOpte51016_LDH_SGA8.1	this study	MZ555486
MOpte51017	n/a	chimpanzee	Pte	05/02/2007	Cameroon	Mfou sanctuary	dried blood spot	M1	*cytB*	956	MOpte51017_SGA20.1	43	MF693445
									asl	837	MOpte51017_ASL_SGA60.1	this study	MZ555479
									ldh	863	MOpte51017_LDH_SGA8.1	this study	MZ927539
									SWGA	23.6 Mb	partial genome	this study	SRR16133635
Ptv_Leo	Leo	chimpanzee	Ptv	14/02/2002	Cote d'Ivoire	Tai Forest	spleen	M1 or M1-like	*cytB*	1,045	n/a	44	GU815517
							spleen, lung	M1-like	SWGA	321 kb	partial genome	this study	SRR16133634

[a]Pb, *P. blacklocki;* Pp, *P. praefalciparum*, Pg, *P. gaboni*, Pr, *P. reichenowi*, Pv, *P. vivax*. References 22, 45, 46 and unpublished data.

Supplementary Table 2. Details of sequences in Fig. 1[a].

Code	Locus	Sequence ID	Host species	Country of origin	Lineage[b]	Accession no.	Reference
1	mtDNA	LOP_P.sp_ An. vinckei 20	mosquito	Gabon	M2	KU318079	2
2	mtDNA	SYptt92_SGA3.1	chimpanzee	Cameroon	M2	MN175636	this study
3	mtDNA	MBptt781_FD_SGA2.1	chimpanzee	Cameroon	M2	MN175639	this study
4	mtDNA	LEK_P.sp_ An. vinckei 17	mosquito	Gabon	M2	KU318080	2
5	mtDNA	LEK_P.sp_ An. vinckei 15	mosquito	Gabon	M2	KU318077	2
6	mtDNA	TL2pp3862_cytb_1.4	bonobo	DRC[c]	M2	KY790535	47
7	mtDNA	LOP_P.sp_ An. vinckei 19	mosquito	Gabon	M2	KU318081	2
8	mtDNA	LEK_P.sp_ An. vinckei 21	mosquito	Gabon	M2	KU318082	2
9	mtDNA	LEK_P.sp_ An. vinckei 18	mosquito	Gabon	M2	KU318073	2
10	mtDNA	SAggg3157_SGA30.55	gorilla	Cameroon	M2	MN175638	this study
11	mtDNA	DGptt540_SGA80.4	chimpanzee	Cameroon	M2	HM234994	48
12	mtDNA	LEK_P.sp_ An. vinckei 14	mosquito	Gabon	M2	KU318076	2
13	mtDNA	LEK_P.sp_ An. vinckei 13	mosquito	Gabon	M2	KU318074	2
14	mtDNA	LEK_P.sp_ An. vinckei 16	mosquito	Gabon	M2	KU318078	2
15	mtDNA	LOP_P.sp_ An. vinckei 12	mosquito	Gabon	M2	KU318075	2
16	mtDNA	GTggg618_SGA10.1	gorilla	Republic of the Congo	M2	MN175637	this study
17	mtDNA	LBptt208_SGA5.2	chimpanzee	Cameroon	M1-like	HM235345	48
18	mtDNA	GA01	chimpanzee	Gabon	M1-like	ERS1452911	1/this study
19	mtDNA	GTggg618_FD_SGA1.2	gorilla	Republic of the Congo	M1-like	MZ555468	this study
20	mtDNA	GA02	chimpanzee	Gabon	M1-like	ERS434571	1/this study
21	mtDNA	*P. brasilianum*	squirrel monkey	Peru	M1	GQ355484	49
22	mtDNA	PmUG01	human	Uganda	M1	LT594637	1
23	mtDNA	SYptt63_SGA20.3	chimpanzee	Cameroon	M1-like	MF693447	43
24	mtDNA	SYptt65_FD_SGA1.1	chimpanzee	Cameroon	M1-like	MZ555469	this study
25	mtDNA	SYpte66_SGA20.2	chimpanzee	Cameroon	M1-like	MF693447	43
26	mtDNA	LBggg1222_SGA5.3	gorilla	Cameroon	M1-like	MF693444	43
27	mtDNA	Oumu	chimpanzee	unknown (West Africa)	M1-like	AB489193	50
28	mtDNA	Takaboh	chimpanzee	Sierra Leone	M1-like	AB489192	50
29	mtDNA	ND-28	human	Cameroon	M1	MF693442	43
30	mtDNA	BI-4	human	Cameroon	M1	MF693433	43
31	mtDNA	Hu111_SGA100.1	human	Cameroon	M1	MF693426	43
32	mtDNA	PmUganda	human	Uganda	M1	AB354570	51
33	mtDNA	PmThailand	human	Thailand	M1	AB489194	50
34	mtDNA	MOpte51016_SGA20.1	chimpanzee	Cameroon	M1	MF693452	43
35	mtDNA	BI-9	human	Cameroon	M1	MF693435	43
36	mtDNA	BI-3	human	Cameroon	M1	MF693441	43

37	mtDNA	DRCQ	bonobo	DRC	M1	GQ355485	49
38	mtDNA	DRCJ	bonobo	DRC	M1	GQ355486	49
39	mtDNA	Hu161_SGA100.8	human	Cameroon	M1	MF693428	43
40	mtDNA	Hu152_SGA100.6	human	Cameroon	M1	MF693427	43
41	mtDNA	Hu115_SGA1000.3	human	Cameroon	M1	MF693425	43
42	*asl*	PGABG03	chimpanzee	Gabon	M2	ERS333073	4/this study
43	*asl*	MBptt781_ASL_SGA4.3	chimpanzee	Cameroon	M2	MZ555470	this study
44	*asl*	GA02	chimpanzee	Gabon	M1-like	ERS434571	1
45	*asl*	PMLGA01_040017600	chimpanzee	Gabon	M1-like	LT594492	1
46	*asl*	SYpte66_ASL_SGA1.1	chimpanzee	Cameroon	M1-like	MZ555477	this study
47	*asl*	SYptt61_ASL_SGA1.1	chimpanzee	Cameroon	M1-like	MZ555476	this study
48	*asl*	MOpte51016_ASL_SGA20.1	chimpanzee	Cameroon	M1	MZ555478	this study
49	*asl*	*P. brasilianum*	squirrel monkey	Bolivia	M1	MKLA02000465	29
50	*asl*	PmUG01_04025800	human	Uganda	M1	LT594625	1
51	*asl*	PMALA_069230	human	Uganda	M1	FLQW01005685	27
52	*asl*	MOpte51017_ASL_SGA60.1	chimpanzee	Cameroon	M1	MZ555479	this study
53	*asl*	PmSingapore	human	Singapore/DRC	M1	KP050474	52
54	*ldh*	MBptt781_LDH_SGA2.9	chimpanzee	Cameroon	M2	MZ555482	this study
55	*ldh*	SYptt92_LDH_SGA4.4	chimpanzee	Cameroon	M2	MZ555480	this study
56	*ldh*	PMALA_021610	human	Uganda	M1	FLQW01001161	27
57	*ldh*	PmUG01_12039800	human	Uganda	M1	LT594633	1
58	*ldh*	*P. brasilianum*	squirrel monkey	Bolivia	M1	MKLA02000540	29
59	*ldh*	MOpte51016_LDH_SGA8.1	chimpanzee	Cameroon	M1	MZ555486	this study
60	*ldh*	MOptt51017_SGA8.1	chimpanzee	Cameroon	M1	MZ555487	this study
61	*ldh*	SYptt63_LDH_SGA2.1	chimpanzee	Cameroon	M1-like	MZ555483	this study
62	*ldh*	SYpte66_LDH_SGA1.1	chimpanzee	Cameroon	M1-like	MZ555483	this study
63	*ldh*	SYpte66_LDH_SGA2.10	chimpanzee	Cameroon	M1-like	MZ555484	this study
64	*ldh*	PMLGA01_120033400	chimpanzee	Gabon	M1-like	LT594500	1
65	*ldh*	SYptt61_LDH_SGA1.3	chimpanzee	Cameroon	M1-like	MZ555483	this study

[a] Published *cytB* sequences not included in these trees are described in Supplementary Table 3.
[b]M1 and M1-like are poorly resolved by *asl* and *ldh* alignments but the most likely lineage is given in each case based on whether sequences are more similar to the M1 (PmUG01) or to the M1-like (PmlGA01) reference genomes.
[c]DRC, Democratic Republic of the Congo

Supplementary Table 3. *CytB* sequences from ape *P. malariae*-related parasites that were not included in the tree in Fig. 1b[a].

Sequence ID	Host species	Country of origin	Accession no.	Reference	Sequence length (bp)	Relationship to sequences in the tree
SYpte66_SGA20.8	chimpanzee	Cameroon	MF693448	43	955	1 nt difference from GA01 (sequence 18)
Ptv_Olduvai	chimpanzee	Cote d'Ivoire	GU815516	44	1,082	2 nt difference from GA02 (sequence 20)
MSggg7212_SGA5.7	gorilla	Cameroon	MF693446	43	954	1 nt difference from GA02 (sequence 20)
TCptt19_SGA600.7	chimpanzee	Republic of the Congo	MF693451	43	956	identical to PmUganda (sequence 32)
Ptv_Leo	chimpanzee	Cote d'Ivoire	GU815517	44	1,045	identical to Hu152_SGA100.6 (sequence 40)
MOpte51017_SGA20.1	chimpanzee	Cameroon	MF693445	43	956	identical to PmUganda (sequence 32)
CPZcam83	chimpanzee	Cameroon	HM000110	53	709	identical to PmUG01 (sequence 22)
Ogooue-CPZ_Dec2013-LEK	chimpanzee	Gabon	KU759796	54	687	identical to PmUG01 (sequence 22)
Caroline-Gor_2011-CDP	gorilla	Gabon	KU759801	54	687	identical to GTggg618_FD_SGA1.2 (sequence 19)
Flore-CPZ_2014-LEK	chimpanzee	Gabon	KU759826	54	687	identical to GA01 (sequence 18)
SYptt65_SGA20.10	chimpanzee	Cameroon	MF693450	43	955	identical to SYptt65_SGA1.1 (sequence 24) and from the same animal

[a]Sequences in this table were not included in Fig. 1b because they did not cover the full length of the 2,038 bp alignment used for the tree.

Supplementary Table 4. Details of M1 and M1-like genomes in the study.

Strain ID	Country of origin	Callable bases[a]	Host	Lineage	Reference	Accession no.	High coverage set?
PmUG01[b]	Uganda	29,539,458	Human	M1	1	GCA_900090045	yes
UAN	Uganda	27,601,126	Human	M1	27	ERS1054709	yes
KEN01	Kenya	21,189,956	Human	M1	28	ERS4423432	yes
LBR02	Liberia	25,193,861	Human	M1	28	ERS4423434	yes
SDN01	Sudan	21,248,019	Human	M1	28	ERS4423436	yes
SLE02	Sierra Leone	24,421,011	Human	M1	28	ERS4423437	yes
SLE04	Sierra Leone	13,524,248	Human	M1	28	ERS4423438	no
THA01	Thailand	12,683,789	Human	M1	28	ERS4423439	no
THA02	Thailand	18,987,182	Human	M1	28	ERS4423440	no
THA05	Thailand	26,180,685	Human	M1	28	ERS4423442	yes
THA06	Thailand	22,291,917	Human	M1	28	ERS4423443	yes
THA09	Thailand	15,271,073	Human	M1	28	ERS4423446	no
THA10	Thailand	25,249,599	Human	M1	28	ERS4423447	yes
THA11	Thailand	19,922,097	Human	M1	28	ERS4423448	no
THA12	Thailand	23,662,004	Human	M1	28	ERS4423449	yes
UGA01	Uganda	14,031,289	Human	M1	28	ERS4423451	no
UGA03	Uganda	24,804,935	Human	M1	28	ERS4423452	yes
UGA07	Uganda	17,983,196	Human	M1	28	ERS4423453	no
GN01	Ghana	28,391,574	Human	M1	1	ERS567899	yes
ID01	India	25,786,888	Human	M1	1	ERS1110321	yes
MA01	Mali	28,098,985	Human	M1	1	ERS1110325	yes
MY01	Malaysia	27,977,920	Human	M1	1	ERS1110317	yes
Pbrasilianum[c]	Bolivia	14,722,663	Squirrel monkey	M1	29	GCA_001885115	no
MOpte51017	Cameroon	23,585,487	Chimpanzee	M1	this study	SRR16133635	yes
Ptv_Leo	Cote d'Ivoire	321,244	Chimpanzee	M1-like	this study	SRR16133634	no
GA01	Gabon	20,352,681	Chimpanzee	M1-like	1	ERS1452911	yes
GA02	Gabon	6,234,616	Chimpanzee	M1-like	1	ERS434571	yes

[a]See Methods for details.
[b]Reference genome.
[c]For *P. brasilianum*, the "callable bases" column gives the number of bases in the reference that could be included in an alignment with *P. brasilianum* contigs.

Supplementary Table 5. Primers used for diagnostic and limiting dilution PCR.

Gene	Amplicon length (kb)	First round primer		Second round primer	
		Name	Sequence	Name	Sequence
cytb	0.6	Pm4659p	5′-ATTTATTATCTTCAATTCCAGCACTT-3′	Pm4740p	5′-ATTACATTTTATACTTCCATTTGTTGC-3′
		Pm5501n	5′-GCATGTTAACTCGATAAATACTAA-3′	Pm5369n	5′-TTCAGAAATATCGTCTTATCGTAGC-3′
	0.8	DW2	5'-TAATGCCTAGACGTATTCCTGATTATCCAG-3'	PLAS1	5'-GAGAATTATGGAGTGGATGGTG-3'
		DW4	5'-TGTTTGCTTGGGAGCTGTAATCATAATGTG-3'	PLAS2a	5'-GTGGTAATTGACATCCWATCC-3'
	1.0	DW2	5'-TAATGCCTAGACGTATTCCTGATTATCCAG-3'	Pfcytb1	5'-CTCTATTAATTTAGTTAAAGCACA-3'
		DW4	5'-TGTTTGCTTGGGAGCTGTAATCATAATGTG-3'	PLAS2a	5'-GTGGTAATTGACATCCWATCC-3'
cox1/cytb	2.5	Pv2768p	5'-GTATGGATCGAATCTTACTTATTC-3'	Pv2856p	5'-CTTATTACAAATTGCAATCATAAAACTTTAGGT-3'
		Pv5739n	5'-AATATCTATAAACCAAATAAAGTKAAACCAATT-3'	Pv5434n	5'-TTCAGAGATATCGTCTTATCGTAGC-3'
asl	0.8	PmAslF1	5'-ATGGAGCACCTGMARAACATCTC-3'	PmAslF2	5'-TCCCATTGACGGTMGGTACARAAAG-3'
		PmAslR1	5'-TGTARRTTCCCYTCTGCATTTTCAAA-3'	PmAslR2	5'-ATTGGGTTRACYTTATGYGGCAT-3'
ldh	0.7	PmLDHF1	5'-ATGGCACCCAAAACAAAAATTGTTC-3'	PmLDHF1	5'-ATGGCACCCAAAACAAAAATTGTTC-3'
		PmLDHR3	5'-GCCTTCATTCTCTTCGTTTCAGC-3'	PmLDHR4	5'-TCATCAAATTTTTTTTTCTCTTC-3'

Supplementary Table 6. SWGA primer sets.

Set	Name	Sequence[a]	Note
**Pm_set37**	Pm_set37-1	AAACGAAAT*A*A	
	Pm_set37-2	AACGAAAAA*T*A	
	Pm_set37-3	AACGTAAAA*A*A	
	Pm_set37-4	ACGAAAAAA*T*A	
	Pm_set37-5	ATACGTAAA*T*A	
	Pm_set37-6	CGAAAAAAA*A*T	
	Pm_set37-7	CGAAAAAAA*T*A	
	Pm_set37-8	TAACGAAAA*A*A	
	Pm_set37-9	TACGAA*C*G	
	Pm_set37-10	TACGCATAT*A*T	
	Pm_set37-11	TACGTAAAA*A*A	
	Pm_set37-12	TATCGAAA*A*A	
	Pm_set37-13	TTTACGAA*T*A	
	Pm_set37-14	TTTCGTAA*T*A	
**Pm_set31**	Pm_set31-1	AAACGAAAT*A*A	=set37-1
	Pm_set31-2	AACGAAAAA*T*A	=set37-2
	Pm_set31-3	ACGAAAAAA*T*A	=set37-4
	Pm_set31-4	ACGTAAAAA*A*A	
	Pm_set31-5	ACGTAAAAA*A*T	
	Pm_set31-6	ATACGTAAA*A*A	
	Pm_set31-7	ATACGTAAA*T*A	=set37-5
	Pm_set31-8	CGAAAAAAA*A*T	=set37-6
	Pm_set31-9	TAACGAAAA*A*A	=set37-8
	Pm_set31-10	TACGAA*C*G	=set37-9
	Pm_set31-11	TACGCATAT*A*T	=set37-10
	Pm_set31-12	TATCGAAA*A*A	=set37-12
	Pm_set31-13	TTTACGAA*T*A	=set37-13
	Pm_set31-14	TTTCGTAA*T*A	=set37-14

[a]Asterisks indicate the location of phosphorothioate bonds necessary to prevent degradation by the phi29 polymerase.

Supplementary Table 7. Start and end of chromosome core regions in PmUG01[a].

Chromosome	Core start	Core end
1	201,469	1,076,788
2	96,669	756,354
3	168,954	1,098,727
4	210,806	1,008,518
5	400,000	1,645,075
6	1	632,997
7	258,480	1,845,043
8	448,181	2,050,031
9	220,459	2,263,000
10	200,871	1,796,872
11	417,858	2,561,830
12	127,109	3,275,472
13	209,966	2,337,377
14	509,643	3,534,179

[a]Core regions and subtelomeres were defined as described in Supplementary Note 2.

Supplementary References

1. Rutledge, G. G. *et al. Plasmodium malariae* and *P. ovale* genomes provide insights into malaria parasite evolution. *Nature* **542**, 101–104 (2017).
2. Makanga, B. *et al.* Ape malaria transmission and potential for ape-to-human transfers in Africa. *Proc. Natl. Acad. Sci. U.S.A.* **113**, 5329–5334 (2016).
3. Brumpt, E. Les parasites du paludisme des chimpanzés. *C. R. Soc. Biol.* **130**, 837–840 (1939).
4. Otto, T. D. *et al.* Genomes of all known members of a *Plasmodium* subgenus reveal paths to virulent human malaria. *Nat. Microbiol.* **3**, 687–697 (2018).
5. Gilabert, A. *et al. Plasmodium vivax*-like genome sequences shed new insights into *Plasmodium vivax* biology and evolution. *PLoS Biol.* **16**, e2006035 (2018).
6. Li, H. & Durbin, R. Fast and accurate short read alignment with Burrows–Wheeler transform. *Bioinformatics* **25**, 1754–1760 (2009).
7. Li, H. Aligning sequence reads, clone sequences and assembly contigs with BWA-MEM. Preprint at https://arxiv.org/abs/1303.3997 (2013).
8. Li, H. *et al.* The Sequence Alignment/Map format and SAMtools. *Bioinformatics* **25**, 2078–2079 (2009).
9. Auburn, S. *et al.* A new *Plasmodium vivax* reference sequence with improved assembly of the subtelomeres reveals an abundance of pir genes. *Wellcome Open Res.* **1**, 4 (2016).
10. Bankevich, A. *et al.* SPAdes: a new genome assembly algorithm and its applications to single-cell sequencing. *J. Comput. Biol.* **19**, 455–477 (2012).
11. Marçais, G. *et al.* MUMmer4: A fast and versatile genome alignment system. *PLoS Comput. Biol.* **14**, e1005944 (2018).
12. Camacho, C. *et al.* BLAST+: architecture and applications. *BMC Bioinform.* **10**, 421 (2009).

13. Assefa, S., Keane, T. M., Otto, T. D., Newbold, C. & Berriman, M. ABACAS: algorithm-based automatic contiguation of assembled sequences. *Bioinformatics* **25**, 1968–1969 (2009).
14. Carver, T. J. *et al.* ACT: the Artemis comparison tool. *Bioinformatics* **21**, 3422–3423 (2005).
15. Tsai, I. J., Otto, T. D. & Berriman, M. Improving draft assemblies by iterative mapping and assembly of short reads to eliminate gaps. *Genome Biol.* **11**, R41 (2010).
16. Otto, T. D., Dillon, G. P., Degrave, W. S. & Berriman, M. RATT: Rapid Annotation Transfer Tool. *Nucleic Acids Res.* **39**, e57–e57 (2011).
17. Aurrecoechea, C. *et al.* PlasmoDB: a functional genomic database for malaria parasites. *Nucleic Acids Res.* **37**, D539–D543 (2009).
18. Abascal, F., Zardoya, R. & Telford, M. J. TranslatorX: multiple alignment of nucleotide sequences guided by amino acid translations. *Nucleic Acids Res.* **38**, W7–W13 (2010).
19. Edgar, R. C. MUSCLE: multiple sequence alignment with high accuracy and high throughput. *Nucleic Acids Res.* **32**, 1792–1797 (2004).
20. Paradis, E., Claude, J. & Strimmer, K. APE: Analyses of Phylogenetics and Evolution in R language. *Bioinformatics* **20**, 289–290 (2004).
21. Pain, A. *et al.* The genome of the simian and human malaria parasite *Plasmodium knowlesi*. *Nature* **455**, 799–803 (2008).
22. Loy, D. E. *et al.* Evolutionary history of human *Plasmodium vivax* revealed by genome-wide analyses of related ape parasites. *Proc. Natl. Acad. Sci. U.S.A.* **115**, E8450–E8459 (2018).
23. Otto, T. D. *et al.* A comprehensive evaluation of rodent malaria parasite genomes and gene expression. *BMC Biol.* **12**, (2014).
24. Gardner, M. J. *et al.* Genome sequence of the human malaria parasite *Plasmodium falciparum*. *Nature* **419**, (2002).
25. Li, L., Stoeckert, C. J. & Roos, D. S. OrthoMCL: identification of ortholog groups for eukaryotic genomes. *Genome Res.* **13**, 2178–2189 (2003).

26. Castresana, J. Selection of conserved blocks from multiple alignments for their use in phylogenetic analysis. *Mol. Biol. Evol.* **17**, 540–552 (2000).
27. Ansari, H. R. *et al.* Genome-scale comparison of expanded gene families in *Plasmodium ovale wallikeri* and *Plasmodium ovale curtisi* with *Plasmodium malariae* and with other *Plasmodium* species. *Int. J. Parasitol.* **46**, 685–696 (2016).
28. Ibrahim, A. *et al.* Selective whole genome amplification of *Plasmodium malariae* DNA from clinical samples reveals insights into population structure. *Sci. Rep.* **10**, 10832 (2020).
29. Talundzic, E. *et al.* First full draft genome sequence of *Plasmodium brasilianum*. *Genome Announc.* **5**, e01566-16 (2017).
30. Joy, D. A. *et al.* Early origin and recent expansion of *Plasmodium falciparum*. *Science* **300**, 318–321 (2003).
31. Pacheco, M. A. *et al.* Timing the origin of human malarias: the lemur puzzle. *BMC Evol. Biol.* **11**, 299 (2011).
32. Auwera, G. A. *et al.* From FastQ data to high-confidence variant calls: the Genome Analysis Toolkit best practices pipeline. *Curr. Protoc. Bioinformatics* **43**, (2013).
33. Danecek, P. *et al.* Twelve years of SAMtools and BCFtools. *GigaScience* **10**, (2021).
34. Page, A. J. *et al.* SNP-sites: rapid efficient extraction of SNPs from multi-FASTA alignments. *Microb. Genom.* **2**, e000056 (2016).
35. Chang, C. C. *et al.* Second-generation PLINK: rising to the challenge of larger and richer datasets. *Gigascience* **4**, 1–16 (2015).
36. Keightley, P. D. & Jackson, B. C. Inferring the probability of the derived vs. the ancestral allelic state at a polymorphic site. *Genetics* **209**, 897–906 (2018).
37. Rand, D. M. & Kann, L. M. Excess amino acid polymorphism in mitochondrial DNA: contrasts among genes from *Drosophila*, mice, and humans. *Mol. Biol. Evol.* **13**, 735–748 (1996).
38. Stoletzki, N. & Eyre-Walker, A. Estimation of the Neutrality Index. *Mol. Biol. Evol.* **28**, 63–70 (2011).

39. MalariaGEN *et al.* An open dataset of Plasmodium falciparum genome variation in 7,000 worldwide samples. *Wellcome Open Res* **6**, 42 (2021).
40. Pearson, R. D. *et al.* Genomic analysis of local variation and recent evolution in Plasmodium vivax. *Nature Genetics* **48**, 959–964 (2016).
41. Sargeant, T. J. *et al.* Lineage-specific expansion of proteins exported to erythrocytes in malaria parasites. *Genome Biology* **7**, R12 (2006).
42. Carver, T., Thomson, N., Bleasby, A., Berriman, M. & Parkhill, J. DNAPlotter: circular and linear interactive genome visualization. *Bioinformatics* **25**, 119–120 (2009).
43. Loy, D. E. *et al.* Investigating zoonotic infection barriers to ape *Plasmodium* parasites using faecal DNA analysis. *Int. J. Parasitol.* **48**, 531–542 (2018).
44. Kaiser, M. *et al.* Wild chimpanzees Infected with 5 *Plasmodium* species. *Emerg. Infect. Dis.* **16**, 1956–1959 (2010).
45. Plenderleith, L. J. *et al.* Adaptive evolution of RH5 in ape *Plasmodium* species of the *Laverania* subgenus. *mBio* **9**, e02237-17 (2018).
46. Sundararaman, S. A. *et al.* Genomes of cryptic chimpanzee *Plasmodium* species reveal key evolutionary events leading to human malaria. *Nat. Commun.* **7**, 11078 (2016).
47. Liu, W. *et al.* Wild bonobos host geographically restricted malaria parasites including a putative new *Laverania* species. *Nat. Commun.* **8**, 1635 (2017).
48. Liu, W. *et al.* Origin of the human malaria parasite *Plasmodium falciparum* in gorillas. *Nature* **467**, 420–425 (2010).
49. Krief, S. *et al.* On the diversity of malaria parasites in African apes and the origin of *Plasmodium falciparum* from bonobos. *PLoS Pathog.* **6**, e1000765 (2010).
50. Hayakawa, T. *et al.* Identification of *Plasmodium malariae*, a human malaria parasite, in imported chimpanzees. *PLoS ONE* **4**, e7412 (2009).
51. Hayakawa, T., Culleton, R., Otani, H., Horii, T. & Tanabe, K. Big Bang in the evolution of extant malaria parasites. *Mol. Biol. Evol.* **25**, 2233–2239 (2008).

52. Chavatte, J.-M., Tan, S. B. H., Snounou, G. & Lin, R. T. P. V. Molecular characterization of misidentified *Plasmodium ovale* imported cases in Singapore. *Malar. J.* **14**, 454 (2015).
53. Duval, L. *et al.* African apes as reservoirs of *Plasmodium falciparum* and the origin and diversification of the *Laverania* subgenus. *Proc. Natl. Acad. Sci. U.S.A.* **107**, 10561–10566 (2010).
54. Ngoubangoye, B. *et al.* The host specificity of ape malaria parasites can be broken in confined environments. *Int. J. Parasitol.* **46**, 737–744 (2016).
