# Supplementary material for: Zoonotic origin of the human malaria parasite Plasmodium malariae from African apes
Source: Nat Commun. 2022 Apr 6;13:1868. doi: 10.1038/s41467-022-29306-4 (PMC8987028; doi:10.1038/s41467-022-29306-4)
Supplement: Supplementary file 2 — Reporting Summary [file 41467_2022_29306_MOESM2_ESM.pdf]

nature portfolio

Corresponding author(s): Lindsey J. Plenderleith

Last updated by author(s): Mar 17, 2022

Reporting Summary

Nature Portfolio wishes to improve the reproducibility of the work that we publish. This form provides structure for consistency and transparency in reporting. For further information on Nature Portfolio policies, see our Editorial Policies and the Editorial Policy Checklist.

Statistics

For all statistical analyses, confirm that the following items are present in the figure legend, table legend, main text, or Methods section.

n/a	Confirmed	
☒	☐	The exact sample size (*n*) for each experimental group/condition, given as a discrete number and unit of measurement
☒	☐	A statement on whether measurements were taken from distinct samples or whether the same sample was measured repeatedly
☒	☐	The statistical test(s) used AND whether they are one- or two-sided<br>*Only common tests should be described solely by name; describe more complex techniques in the Methods section.*
☒	☐	A description of all covariates tested
☒	☐	A description of any assumptions or corrections, such as tests of normality and adjustment for multiple comparisons
☒	☐	A full description of the statistical parameters including central tendency (e.g. means) or other basic estimates (e.g. regression coefficient) AND variation (e.g. standard deviation) or associated estimates of uncertainty (e.g. confidence intervals)
☒	☐	For null hypothesis testing, the test statistic (e.g. *F*, *t*, *r*) with confidence intervals, effect sizes, degrees of freedom and *P* value noted<br>*Give P values as exact values whenever suitable.*
☒	☐	For Bayesian analysis, information on the choice of priors and Markov chain Monte Carlo settings
☒	☐	For hierarchical and complex designs, identification of the appropriate level for tests and full reporting of outcomes
☒	☐	Estimates of effect sizes (e.g. Cohen's *d*, Pearson's *r*), indicating how they were calculated

Our web collection on statistics for biologists contains articles on many of the points above.

Software and code

Policy information about availability of computer code

Data collection	No software was used in data collection.
Data analysis	All software used is described in the Methods and Supplementary Information. Commercial software used for data analysis was Geneious (version 20202.1.2). All other software is publicly available: ABACAS (version 1), ACT (version 18.0.2), ape (version 5.4.1), blastn (version 2.9.0), bcftools (version 1.9), bwa (version 0.7.12), Clustal Omega (version 1.2.3), cutadapt (version 1.9.1), Dustmasker (version 1.0.0), est-sfs unfolder (version 2.03), GATK (version 4.1.6.0), Gblocks (version 0.91b), IMAGE (version 2.4), MUMmer (4.0.0beta2), MUSCLE (version 3.8.31), OrthoMCL (version 2), PlasmoDB (release 55), plink (version 1.9), RATT (version 1), RAxML (version 8.2.12), SAMtools (version 1.9), smalt (version 0.6.7), snp-sites (version 1), SPades (version 3.14.1), SplitsTree4 (version 4.16.1), TranslatorX (version 1.1).

For manuscripts utilizing custom algorithms or software that are central to the research but not yet described in published literature, software must be made available to editors and reviewers. We strongly encourage code deposition in a community repository (e.g. GitHub). See the Nature Portfolio guidelines for submitting code & software for further information.

Data

Policy information about availability of data

All manuscripts must include a data availability statement. This statement should provide the following information, where applicable:

- Accession codes, unique identifiers, or web links for publicly available datasets
- A description of any restrictions on data availability
- For clinical datasets or third party data, please ensure that the statement adheres to our policy

The new sequence data reported in this paper have been deposited in the appropriate NCBI databases under accession numbers MN175636-MN175639, MZ555468-MZ555486, MZ927539 (GenBank, SGA sequences) and PRJNA767638 (Sequence Read Archive, SWGA sequences), as noted in Supplementary Table 1.

Scaffolds from the new M2 assembly derived from sequence library ERS333073 are available in the Third Party Annotation Section of the DDBJ/ENA/GenBank databases under the accession numbers TPA: BK061131-BK061144. The study also analysed publicly available data from BioProjects PRJEB14392 and PRJNA344798 (genome assemblies), and BioProjects PRJEB13344, PRJEB12680 and PRJEB37746 (read libraries), details in Supplementary Table 4.

Field-specific reporting

Please select the one below that is the best fit for your research. If you are not sure, read the appropriate sections before making your selection.

- [x] Life sciences
- [ ] Behavioural & social sciences
- [ ] Ecological, evolutionary & environmental sciences

For a reference copy of the document with all sections, see nature.com/documents/nr-reporting-summary-flat.pdf

Life sciences study design

All studies must disclose on these points even when the disclosure is negative.

Sample size	The number of samples included was dependent on the number of biological samples collected.
Data exclusions	No data were excluded.
Replication	Not applicable because no experiments were performed.
Randomization	Not applicable because the study did not include experimental comparison of different groups.
Blinding	Not applicable because the study did not include experimental comparison of different groups.

Reporting for specific materials, systems and methods

We require information from authors about some types of materials, experimental systems and methods used in many studies. Here, indicate whether each material, system or method listed is relevant to your study. If you are not sure if a list item applies to your research, read the appropriate section before selecting a response.

Materials & experimental systems

n/a	Involved in the study	
☒	☐	Antibodies
☒	☐	Eukaryotic cell lines
☒	☐	Palaeontology and archaeology
☐	☒	Animals and other organisms
☒	☐	Human research participants
☒	☐	Clinical data
☒	☐	Dual use research of concern

Methods

n/a	Involved in the study	
☒	☐	ChIP-seq
☒	☐	Flow cytometry
☒	☐	MRI-based neuroimaging

Animals and other organisms

Policy information about studies involving animals; ARRIVE guidelines recommended for reporting animal research

Laboratory animals	The study did not involve laboratory animals.
Wild animals	All studies were performed using remnant DNA from blood or fecal samples collected previously from both wild-caught and captive apes for molecular epidemiological studies of SIV and Plasmodium infections. Samples were selected from these existing specimen banks based on their geographic origin, individual information, sample availability and specimen quality.
Field-collected samples	The study did not involve live samples collected from the field.
Ethics oversight	All samples were selected from existing specimen banks. At the time, all samples were obtained with the approval of the respective Institutional Animal Care and Use Committees and samples were shipped in compliance with Convention on International Trade in Endangered Species of Wild Fauna and Flora regulations and country-specific import and export permits as has been published previously (and is summarized in Table S1 and the methods).

Note that full information on the approval of the study protocol must also be provided in the manuscript.
